# Sex differences in the latent structure of suicide risk among patients with mood disorders: taxometric analyses using the ideation-to-action framework

**DOI:** 10.1017/S0033291726104255

**Published:** 2026-04-28

**Authors:** Chanhee Park, Eunbyeol Lee, Myeongkeun Cho, C. Hyung Keun Park

**Affiliations:** 1 Asan Institute for Life Sciences, Asan Medical Center, Seoul, Republic of Korea; 2 Department of Psychiatry, Asan Medical Center, Seoul, Republic of Korea

**Keywords:** ideation-to-action framework, IMV model, sex difference, suicidal behavior, suicide risk, taxometric analysis

## Abstract

**Background:**

Previous taxometric studies have yielded inconsistent findings regarding the empirical support for the common clinical practice of categorizing patients into discrete suicide risk groups (low versus high risk). Furthermore, potential sex differences in these latent structures have not been adequately explored. This study aimed to investigate the latent structure of suicide risk based on motivational and volitional phase symptoms from the ideation-to-action framework, and to explore potential sex differences in these latent structures, in order to determine whether the clinical practice of categorizing patients into low versus high suicide risk categories is empirically valid.

**Methods:**

We employed taxometric procedures to examine whether suicide risk should be understood as dimensional or categorical. Our analysis distinctly evaluated motivational and volitional phase symptoms across separate samples of male and female outpatients with mood disorders.

**Results:**

Our research revealed significant sex differences in the latent structure of suicide risk. For motivational phase symptoms, an ambiguous structure was revealed in the male group, whereas a clearly dimensional latent structure was observed in the female group. For volitional phase symptoms, a categorical structure emerged in males, while a dimensional structure was found in females.

**Conclusions:**

Given the ‘gender paradox’ in suicidal behavior, which highlights higher rates of fatal suicide attempts among males, early identification of the high-volitional-risk group and focused allocation of intervention resources are particularly crucial for males. Our findings underscore the necessity for sex-specific approaches to suicide risk assessments, research applying the ideation-to-action framework, and targeted intervention development.

## Introduction

Suicide constitutes a significant public health concern that results in substantial societal costs and extreme psychological distress among those bereaved by suicide. Given that most psychiatric disorders serve as major risk factors for suicide (Bachmann, 2018), patients with psychiatric disorders typically undergo suicide risk assessments in clinical settings (Rudd, 2023). Due to the limited resources for treatment and prevention, clinicians often classify individuals into high-risk versus low-risk groups to concentrate these resources on those at higher risk for suicide (Witte et al., 2017). To ensure that this distinction is evidence-based, it is essential to determine whether individual differences in suicide risk should be conceptualized dimensionally or categorically (Siddaway et al., 2021; Witte et al., 2017). If the latent structure of suicide risk is dimensional, then individual differences reflect primarily quantitative degrees, suggesting that high- versus low-risk classifications may be somewhat arbitrary (Haslam et al., 2020; Ruscio et al., 2013). In contrast, if the latent structure is categorical, individual differences primarily reflect qualitative distinctions, implying that such risk categorizations could be valid (Haslam et al., 2020; Ruscio et al., 2013).

Taxometric analysis is a key methodological approach for determining the latent structure of a construct. Taxometric procedures employ various mathematically nonredundant analytic techniques to examine whether a categorical boundary differentiating distinct groups exists (Ruscio et al., 2010). To date, six taxometric analyses of suicide risk have been published (Siddaway et al., 2021). However, a meta-analysis synthesizing these findings indicated that the results of taxometric analyses on suicide risk remain ambiguous (Siddaway et al., 2021). Two studies suggested that suicide risk is categorical (Rufino et al., 2018; Witte et al., 2017), whereas the remaining four suggested that suicide risk is dimensional (Liu et al., 2015; Orlando, Broman-Fulks, Whitlock, Curtin & Michael, 2015; Siddaway et al., 2021). In accordance with Siddaway et al. (2021), we propose two possible explanations for the incongruence in previous results. First, this discrepancy may arise because the latent structure of suicidal ideation differs from that of suicidal action. Contemporary suicide theories [interpersonal theory (Van Orden et al., 2010), integrated motivational-volitional (IMV) model (O’Connor & Kirtley, 2018), and three-step theory (Klonsky & May, 2015)] suggest that the manifestation of suicidal ideation and the transition from ideation to attempts are discrete processes that require distinct explanations (Klonsky et al., 2018). This conceptual framework, referred to as the ‘ideation-to-action framework’ has been argued by Klonsky and May (2015) as a foundational approach for suicide research. However, previous research has not fully addressed the potential differences in the latent structure between the factors contributing to suicidal ideation development and those governing the transition from ideation to attempts. Studies have either conducted taxometric analyses using indicators exclusively related to suicidal ideation or have simultaneously incorporated both ideation and action components within a single taxometric analysis (Siddaway et al., 2021).

Second, the latent structure of suicide risk might vary across particular subpopulations, especially with respect to sex. Sex differences in suicidal behavior and associated risk factors have been consistently documented in the literature (Hawton, 2000; Schrijvers et al., 2012). Nevertheless, previous taxometric analyses have not examined potential sex differences. Notably, studies suggesting a dimensional structure of suicide risk were all conducted with samples comprising more than 70% females (Liu et al., 2015; Orlando et al., 2015; Siddaway et al., 2021), while one of the two studies suggesting a categorical structure utilized a sample with more than 70% male participants (Witte et al., 2017). Considering the evident differences in sample composition with respect to sex in previous studies, further investigation is warranted to determine whether sex differences exist in the latent structure of suicide risk (Siddaway et al., 2021).

Therefore, this study aimed to examine the latent structure of suicide risk in outpatients with mood disorders. To apply the ideation-to-action framework to the taxometric analysis of suicide risk, we utilized the integrated motivational-volitional (IMV) model. The IMV model is a tripartite framework that conceptualizes the suicidal process as consisting of three phases: the biopsychosocial context facilitating the emergence of suicidal ideation and behavior (pre-motivational phase), factors contributing to the development of suicidal ideation (motivational phase), and factors governing the progression from suicidal ideation to suicide attempts or death by suicide (volitional phase). The IMV model encompasses diverse risk factors contributing to suicide risk and has received consistent empirical support (O’Connor & Kirtley, 2018). We conducted separate taxometric analyses for factors within the motivational and volitional phases. Additionally, we sought to examine potential sex differences in the latent structure of suicide risk by comparing the findings between males and females.

## Methods

### Participants and procedure

In this study, we used clinical assessment data obtained during standardized intake procedures at the Mood Disorder and Suicide Prevention Clinic and the Obsessive-Compulsive Disorder Clinic within the Department of Psychiatry, Asan Medical Center, a tertiary hospital located in Seoul, Republic of Korea. The data of outpatients who met the following inclusion criteria were included in the analysis: (a) a clinical diagnosis of mood disorders (outpatients diagnosed with unspecified mood disorders were excluded); (b) absence of medical instability; (c) completion of all assessments; and (d) proficiency in the Korean language to ensure valid responses. To utilize the lifetime number of suicide attempts as taxometric indicators, data from outpatients who provided imprecise responses to the suicide attempt question (e.g. ‘I don’t know’ or ‘I cannot remember’) or who reported self-injury with unclear suicidal intent were excluded. Data from 1,052 outpatients (384 males and 668 females; *M_age_* = 28.89, *SD_age_* = 8.47) who presented to the clinic between February 2022 and February 2025 were included in the analysis. Medical records were reviewed by the principal investigator. The authors assert that all procedures contributing to this work comply with the ethical standards of the relevant national and institutional committees on human experimentation and with the Helsinki Declaration of 1975, as revised in 2013. The study protocol was approved by the Institutional Review Board of Asan Medical Center (2025–0445). The requirement for informed consent was waived because of the retrospective nature of the data analysis.

## Measures

### Indicators for taxometric analyses

Based on the IMV model of suicidal behavior (O’Connor & Kirtley, 2018), the latent structures of the motivational and volitional phase symptoms were examined separately. According to previous research (De Beurs et al., 2017; O’Connor & Kirtley, 2018), we classified the variables available in our dataset into motivational or volitional phase symptoms, then selected the final indicator set based on established taxometric data prerequisites (Meehl, 1995; Ruscio et al., 2010). Specifically, taxometric indicators must exhibit standardized mean differences above 1.25 between the taxon (hypothesized discrete subgroup of interest) and complement (remaining individuals not in the taxon), and correlations between indicators within each group must remain below 0.30.

For motivational phase symptoms, perceived burdensomeness, suicidal ideation duration in the past month (the most severe type), and current suicidal ideation were selected as the indicators. Owing to the high correlation between the perceived burdensomeness sum score and suicidal ideation, we utilized the score from a single item of the six items in the Interpersonal Needs Questionnaire (INQ) measuring perceived burdensomeness as a motivational phase indicator (Van Orden et al., 2012). The item containing the word ‘death’ (‘These days I think my death would be a relief to the people in my life’) was selected because of its conceptual closeness to ‘suicide’. The most severe suicidal ideation duration in the past month was measured using a single item (‘When you have the thoughts, how long do they last?’) from the Columbia Suicide Severity Rating Scale (C-SSRS) (Posner et al., 2011), while current suicidal ideation was evaluated with Item 9 of the Patient Health Questionnaire (PHQ-9) (Spitzer et al., 1999), in accordance with previous research (Rufino et al., 2018).

To conduct taxometric analysis for volitional phase symptoms, deterrents of suicidal behavior, current suicide planning, and lifetime number of suicide attempts were selected as indicators (De Beurs et al., 2017; O’Connor & Kirtley, 2018). Deterrents of suicidal behavior were measured using a single item from the C-SSRS: ‘(When the suicidal ideation was most severe in the past month) Are there things – anyone or anything (e.g., family, religion, pain of death) – that stopped you from wanting to die or acting on thoughts of committing suicide?’ Current suicide planning was measured using the second item of the Depression Symptom Inventory-Suicidality Subscale (DSI-SS) (Joiner et al., 2002), and the lifetime number of suicide attempts was assessed with the question, ‘If you have ever attempted suicide in your lifetime, please indicate the number of attempts.’ Consistent with previous taxometric analysis (Witte et al., 2017), participants reporting six or more lifetime suicide attempts were reassigned to have a score of 6 to manage substantial skew.

### Follow-up external validity measures

#### Patients Health Questionnaire-9 (PHQ-9)

The PHQ-9, a validated self-report instrument, evaluates depressive symptoms across a two-week period using the nine diagnostic criteria for depressive disorder (Kroenke et al., 2001). Items are rated on a 4-point Likert scale. This study utilized the Korean-validated version of the PHQ-9, which exhibited good internal consistency in both male and female samples (Park et al., 2010). The internal consistencies in both males and females were acceptable, with Cronbach’s *α* coefficients of 0.89 and 0.84, respectively.

#### Generalized anxiety disorder-7 (GAD-7)

The GAD-7 was used to assess anxiety symptom severity (Spitzer et al., 2006) This validated 7-item self-report measure requires respondents to rate symptom severity during the two-week period prior to the assessment. Participant responses are recorded using a 4-point Likert scale. In the current study, the Korean-validated version of the GAD-7 showed robust internal consistencies in both males and females, with a Cronbach’s *α* coefficient of 0.90 and 0.89 (Lee et al., 2022).

#### Young Schema Questionnaire-Short Form, Version 3 (YSQ-SF-3)

Early maladaptive schemas (EMSs) were measured using the YSQ-SF-3, a self-report instrument comprising 90 items (Young & Brown, 2005). The instrument assesses 18 distinct EMSs: emotional deprivation, abandonment, mistrust/abuse, social isolation/alienation, defectiveness/shame, failure, dependence/incompetence, vulnerability to harm or illness, enmeshment/undeveloped self, subjugation, self-sacrifice, emotional inhibition, unrelenting standards/hypercriticalness, entitlement/grandiosity, insufficient self-control/self-discipline, approval-seeking/recognition seeking, negativity/pessimism, and punitiveness. In the present study, we utilized only the four schemas (social isolation/alienation, defectiveness/shame, failure, and dependence/incompetence) that have been reported to have at least moderate correlations with suicidal ideation in previous meta-analysis as external variables (Pilkington et al., 2021). All four schemas showed strong internal consistency in our sample (Cronbach’s *α* coefficients of 0.84–0.91 in males, 0.83–0.93 in females).

#### Interpersonal Needs Questionnaire-15 (INQ-15)

The INQ-15 was used to assess perceived burdensomeness and thwarted belongingness (Park & Kim, 2019; Van Orden et al., 2012). The instrument comprises two distinct subscales: a 6-item scale measuring perceived burdensomeness and a 9-item scale measuring thwarted belongingness. Both subscales exhibited strong internal consistency in our male and female samples (Cronbach’s *α* coefficients of 0.94 and 0.85 for the perceived burdensomeness and thwarted belongingness subscales in male, 0.94 and 0.84 in female).

#### Acquired Capability for Suicide Scale-Fearlessness About Death (ACSS-FAD)

The ACSS-FAD was used to assess fearlessness about death (Ribeiro et al., 2014; Ryu & You, 2017). In the present study, the total seven items of the ACSS-FAD demonstrated acceptable internal consistency in both males (Cronbach’s *α* = .83) and females (Cronbach’s *α* = .81).

### Statistical analysis

The study employed three nonredundant taxometric procedures: Mean Above Minus Below A Cut (MAMBAC) (Meehl & Yonce, 1994), MAXimum EIGenvalue (MAXEIG) (Waller & Meehl, 1998), and Latent Mode (L-Mode) (Waller & Meehl, 1998). These analyses were conducted using the RTaxometrics package in R 4.4.1 (R Foundation, Vienna, Austria), which incorporates advanced taxometric procedures and analytic strategies.

Each procedure generates distinctive curve patterns for taxonic constructs: inverse U-shaped curves for MAMBAC and MAXEIG, and bimodal curves for L-Mode. However, because curve shapes can be influenced by factors beyond the latent structure (e.g., skewness and indicator associations), simulated datasets that reproduce the essential features of the research data while varying their latent structure were generated for comparison. This comparison was quantified using the comparison curve fit index (CCFI), where values below 0.45 support a dimensional structure, values above 0.55 indicate a taxonic structure, and values between 0.45 and 0.55 suggest ambiguous results (Ruscio et al., 2010).

The analyses employed 10 internal replications for both MAMBAC and MAXEIG to mitigate the issues with tied scores. Additionally, the CCFI profile method was utilized, which generates taxonic comparison data across base rates from 0.025 to 0.975 in 0.025 increments. For taxonic constructs, the CCFI value should peak at the taxon’s true base rate. This method has shown superior accuracy in base rate estimation compared to alternatives (Ruscio et al., 2018). If our taxometric analyses revealed a categorical structure, we employed the CCFI profile method to determine the taxon base rates for subsequent analyses.

Following the identification of a taxonic structure, we conducted further examinations comparing taxon and complement members on external variables that measure various psychological (e.g., depressive symptoms, anxiety symptoms) and suicide risk factors (e.g., perceived burdensomeness, thwarted belongingness). We used independent *t*-tests to calculate the effect sizes for these external measures, thereby evaluating the construct validity of the identified taxon. Follow-up analyses were conducted using SPSS 30.0 (IBM, Armonk, NY).

## Results

### Comparison between male and female outpatients

Table 1 presents demographic and clinical characteristics of male and female outpatients, along with sex differences in taxometric indicators and external variables. The age range was 18–49 years for both sexes. The median age was 26 years for males and 28 years for females. All taxometric indicators, except for current deterrents of suicidal behavior, were significantly higher in females. Among external variables, females exhibited higher levels of depressive symptoms and fearlessness about death compared to males, while thwarted belongingness was significantly higher in males.

**Table 1.** Demographic and clinical characteristics in each sample

	Male(*n* = 384)		Female(*n* = 668)				
Diagnoses, *n*(%)							
Major depressive disorder	140 (36.4)		231 (34.6)				
Bipolar I disorder	59 (15.4)		110 (16.5)				
Bipolar II disorder	134 (34.9)		253 (37.9)				
Other specified depressive disorders	4 (1.0)		7 (1.0)				
Other specified bipolar and related disorder	47 (12.2)		67 (10.0)				

Characteristics	*M*	*SD*	*M*	*SD*	*t*	*df*	*p*
Age	27.79	8.60	29.52	8.34	3.21***	1050	<.001
Depressive symptoms	14.93	6.23	15.84	5.88	2.32*	1050	.02
Anxiety symptoms	12.69	5.71	12.98	5.80	0.80	1050	.43
Perceived burdensomeness	19.46	11.61	20.82	11.83	1.83	1050	.07
Thwarted belongingness	39.68	11.11	38.26	11.60	−1.95*	1050	.05
Fearlessness about death	15.08	6.93	16.38	6.96	2.91**	1050	.004
Motivational taxometric indicators							
Current suicidal ideation	1.39	1.16	1.70	1.17	4.16***	1050	<.001
My death would be a relief	3.42	2.19	3.80	2.34	2.54^*^	1050	.01
Suicidal ideation duration in the past month	1.82	1.45	2.12	1.53	3.24***	1050	<.001
Volitional taxometric indicators							
Current deterrents of suicidal behavior	1.24	1.32	1.36	1.31	1.39	1050	.16
Current suicide planning	1.10	0.93	1.27	0.96	2.87**	1050	.004
Lifetime number of suicide attempts	1.13	1.64	1.55	1.87	3.74***	1050	<.001

*p < .05; **p < .01; ***p < .001.

### Taxometric analyses

Taxometric analyses require indicators demonstrating at least 1.25 standard deviation units of separation between the taxon and complement groups (Meehl, 1995). In our sample, 47% of males and 56% of females had a lifetime history of suicide attempts. Based on one of the key premises of the IMV model (O’Connor & Kirtley, 2018) – that individuals with a history of suicide attempts exhibit higher motivational and volitional phase symptoms compared to those without such history – these base rates were used to compute indicator validity values. All indicators in both samples met the taxometric prerequisites (Supplementary Table 1) (Ruscio et al., 2013).

#### Motivational phase indicators

**Figure 1.**
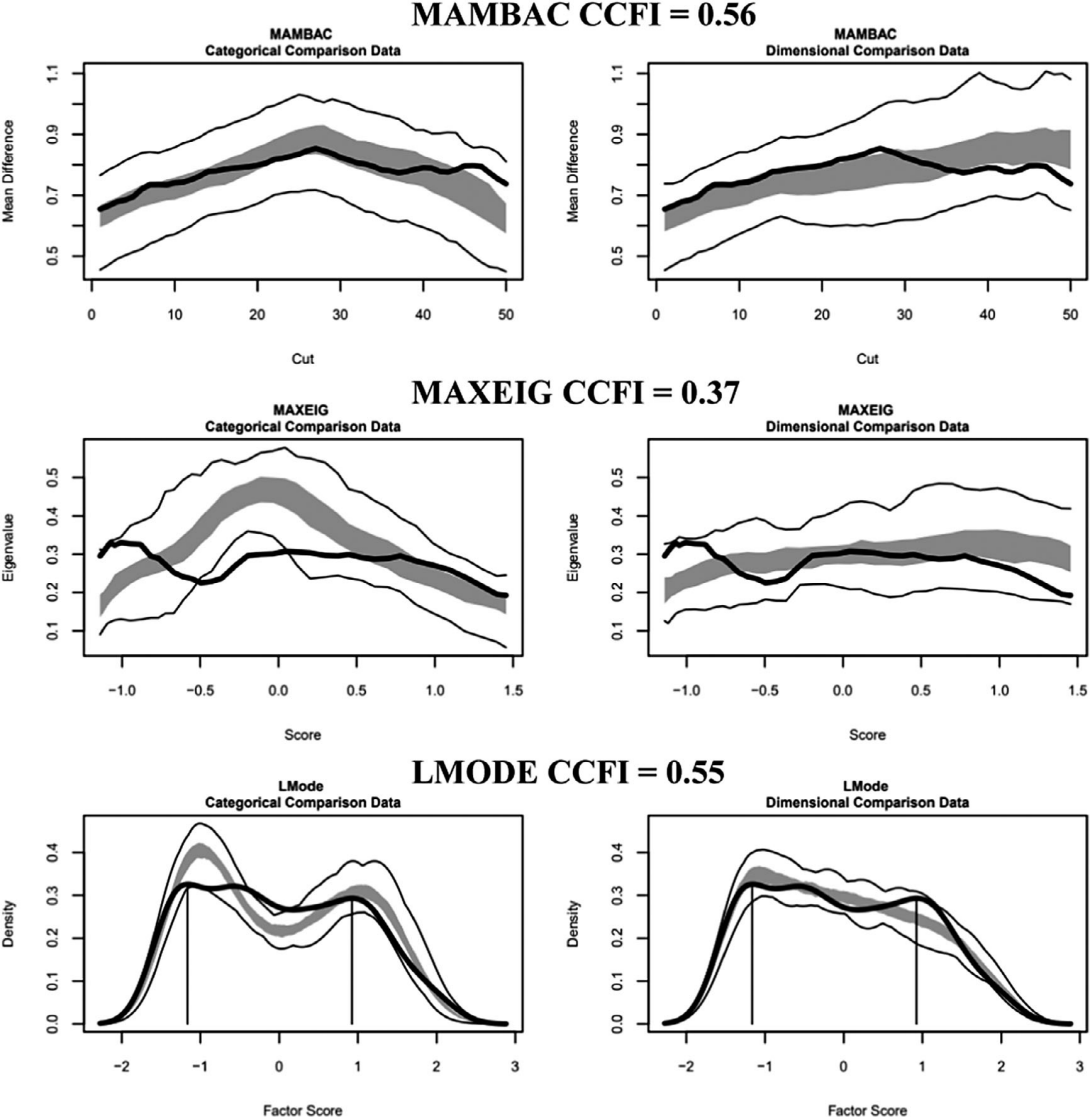
Three taxometric analyses using motivational indicators conducted on the male outpatients. *Note*: Empirical data curves are represented by dark lines, while the boundaries of the comparative analyses are delineated by lighter lines, derived from 100 parallel comparison data samples. The interquartile range (middle 50%) of the values obtained from the parallel comparison data analyses is indicated by the shaded regions. CCFI: comparison curve fit index; L-Mode: Latent mode; MAMBAC: Mean above minus below a cut; MAXEIG: MAXimum EIGenvalue.

**Figure 2.**
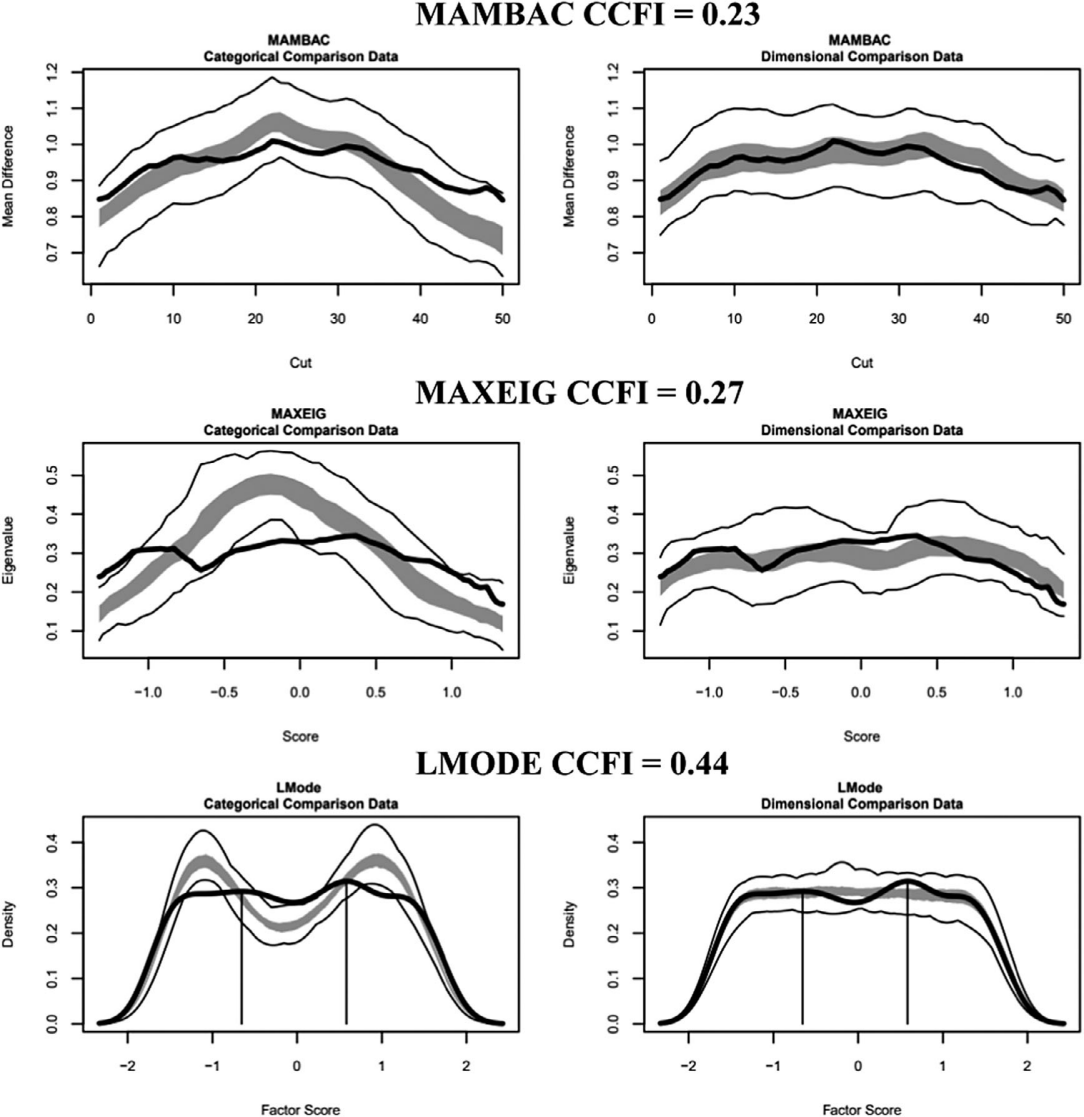
Three taxometric analyses using motivational indicators conducted on the female outpatients. *Note*: Empirical data curves are represented by dark lines, while the boundaries of comparative analyses are delineated by lighter lines, derived from 100 parallel comparison data samples. The interquartile range (middle 50%) of values obtained from the parallel comparison data analyses is indicated by the shaded regions. CCFI: comparison curve fit index; L-Mode: Latent mode; MAMBAC: Mean above minus below a cut; MAXEIG: MAXimum EIGenvalue.

For males, the results were ambiguous, with CCFI values of 0.56 (MAMBAC), 0.37 (MAXEIG), and 0.55 (L-Mode), averaging 0.49. The CCFI profile method yielded 0.45, indicating an ambiguous latent structure (Figure 1, Supplementary Figure 1). For females, the results supported dimensionality with CCFI values of 0.23 (MAMBAC), 0.27 (MAXEIG), and 0.44 (L-Mode), averaging 0.31. The CCFI profile method yielded 0.28, supporting the dimensional structure (Figure 2, Supplementary Figure 2).

#### Volitional phase indicators

**Figure 3.**
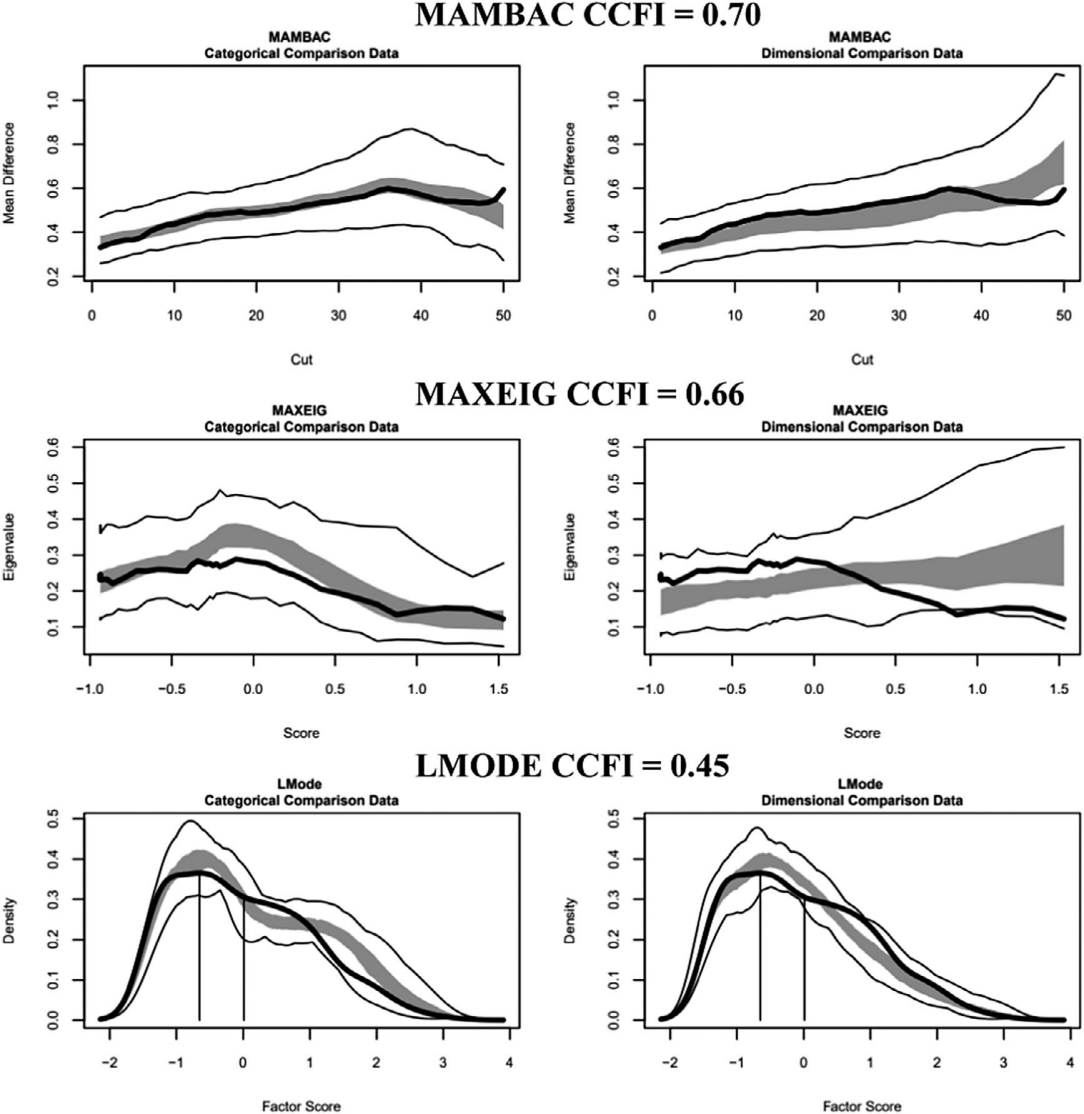
Three taxometric analyses using volitional indicators conducted on the male outpatients. *Note*: Empirical data curves are represented by dark lines, while the boundaries of comparative analyses are delineated by lighter lines, derived from 100 parallel comparison data samples. The interquartile range (middle 50%) of values obtained from the parallel comparison data analyses is indicated by the shaded regions. CCFI: comparison curve fit index; L-Mode: Latent mode; MAMBAC: Mean above minus below a cut; MAXEIG: MAXimum EIGenvalue.

**Figure 4.**
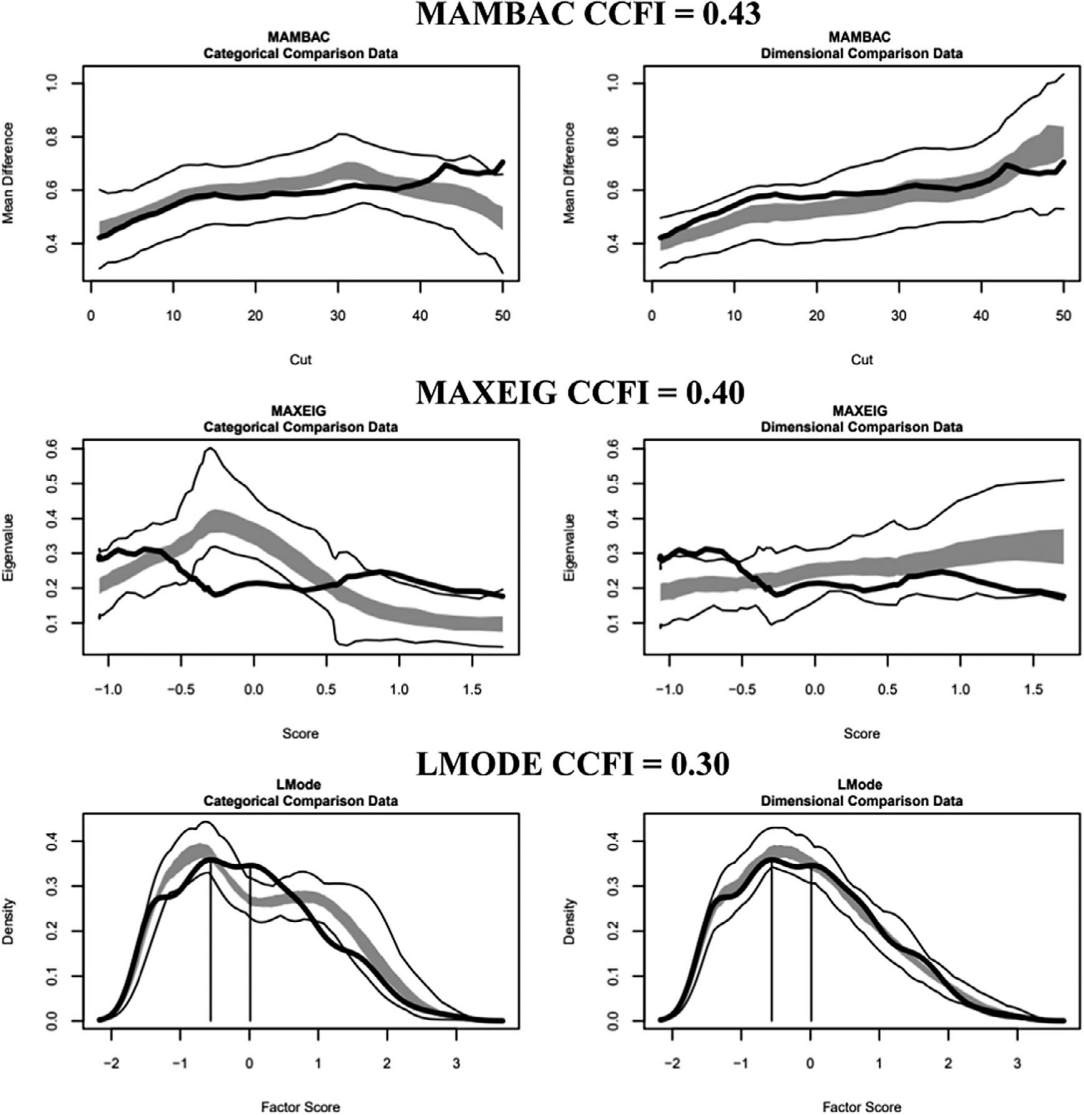
Three taxometric analyses using volitional indicators conducted on the female outpatients. *Note*: Empirical data curves are represented by dark lines, while the boundaries of comparative analyses are delineated by lighter lines, derived from 100 parallel comparison data samples. The interquartile range (middle 50%) of values obtained from the parallel comparison data analyses is indicated by the shaded regions. CCFI: comparison curve fit index; L-Mode: Latent mode; MAMBAC: Mean above minus below a cut; MAXEIG: MAXimum EIGenvalue.

For males, the results indicated taxonicity with CCFI values of 0.70 (MAMBAC), 0.66 (MAXEIG), and 0.45 (L-Mode), averaging 0.60. The CCFI profile method yielded 0.58 and a base rate of 0.63, which was used for external validity analyses, as this method provides more accurate base rate estimates when a taxon exists (Figure 3, Supplementary Figure 3) (Ruscio et al., 2018). For females, the results supported dimensionality with CCFI values of 0.43 (MAMBAC), 0.40 (MAXEIG), and 0.30 (L-Mode), averaging 0.38. The CCFI profile method yielded a value of 0.41 (Figure 4, Supplementary Figure 4).

### Follow-up analyses

To examine the construct validity of the male volitional risk taxon, we compared the taxon members to the complement members on external variables (Supplementary Table 2). As expected, the effect sizes for the external measures were mostly above moderate and sometimes very large (Cohen’s *d*s from 0.30 to 1.04). Large effect sizes were observed in depressive symptoms including suicidal ideation (Cohen’s *d* = 0.70), perceived burdensomeness (Cohen’s *d* = 1.04), and defectiveness/shame schema (Cohen’s *d* = 0.73), while relatively smaller effect sizes were found in anxiety symptoms (Cohen’s *d* = 0.36) and fearlessness about death (Cohen’s *d* = 0.30).

### Sensitivity analyses

To examine whether the observed sex differences in latent structure were attributable to diagnostic or demographic confounds, we conducted two sensitivity analyses. First, we performed taxometric analyses separately for depressive and bipolar disorders (without sex stratification, due to insufficient subsample sizes). Both motivational and volitional phase symptoms showed similar patterns across diagnoses (motivational: dimensional; volitional: ambiguous), suggesting that diagnostic category may not account for the sex differences observed in the main analyses. Second, to control for age differences between male and female participants, we randomly sampled a subset of female outpatients matched to the male outpatients for sample size and age. The dimensional structure of both motivational and volitional phase symptoms in female patients persisted after age and sample size matching. The full results of sensitivity analyses are presented in the Supplementary Materials.

## Discussion

We examined the latent structures of both motivational (related to the development of suicidal ideation) and volitional (related to the progression from ideation to attempts) phase symptoms among males and females with mood disorders. The results revealed significant sex differences. The latent structure of motivational phase symptoms was dimensional in females, but ambiguous in males. The results suggested that the latent structure of volitional phase symptoms was categorical in the male outpatients but dimensional in the female outpatients. Follow-up analyses revealed that male outpatients classified in the high-volitional-risk taxon exhibited significantly higher scores on all external measures compared to those in the low-risk group. Most effect sizes were medium or greater, with particularly large effect sizes observed for perceived burdensomeness. Sensitivity analyses provided further support for these sex-specific findings. When stratified by diagnosis only, motivational phase symptoms were dimensional and volitional phase symptoms were ambiguous in both depressive disorders and bipolar disorders, suggesting that distinct sex-specific structures may be masked when data are pooled across sexes. Additionally, after matching female outpatients to male outpatients for sample size and age, the dimensional structure in females persisted, indicating that age differences did not confound the observed sex differences.

Although a previous study suggested that individual differences in motivational symptoms may be primarily quantitative rather than qualitative (Liu et al., 2015), our study found that this hypothesis was supported only in females. Additionally, our study revealed that the latent structure of motivational phase symptoms in males was ambiguous, showing sex differences in the latent structure of motivational phase symptoms. These findings suggest that categorizing individuals into high- and low-risk suicide groups based solely on indicators related to suicidal ideation lacks empirical support (Siddaway et al., 2021).

However, for the volitional phase symptoms related to actually enacting suicidal behaviors, these factors exhibited a categorical latent structure in males. This suggests that there may be a distinct subgroup of males who are at elevated risk for suicidal behavior enaction. In females, these symptoms remained dimensional, suggesting that risk for suicidal behavior enaction among females still exists on a continuum without clear distinct boundaries. The sex differences in the latent structure of action-related volitional phase symptoms could have several interpretations. First, it aligns with the ‘gender paradox’ in suicidal behavior, which refers to the phenomenon that males tend to use more lethal means and have higher death by suicide rates, despite females having higher rates of suicidal ideation and suicide attempts (Canetto & Sakinofsky, 1998; Schrijvers et al., 2012). Based on the gender paradox in suicide, it has been suggested that the duration of the suicidal process may be shorter in males than in females because once the suicidal process has started, males may have greater difficulty in reversing their decision to act on suicidal ideation than females (Neeleman et al., 2004; Schrijvers et al., 2012; Van Heeringen et al., 2000). In this ‘male suicidal process’, males may often tend to disregard their social resources as deterrents to suicidal behavior, develop more concrete suicide plans, and rapidly engage in suicide attempts (Schrijvers et al., 2012). This pattern may contribute to a prominent distinction between those who have decided to attempt suicide and those who have not decided to act on suicidal ideation in males. Furthermore, given that males tend to use more violent and lethal methods in suicide attempts (Canetto & Sakinofsky, 1998), the high volitional risk group in male outpatients may be more likely to attempt suicide using violent methods. Although the current study did not assess the lethality of suicide attempt methods, this possibility warrants investigation in future research. In contrast, females may reconsider their decision to act on suicidal ideation multiple times (Schrijvers et al., 2012), thereby developing volitional phase symptoms more gradually. A more gradual progression through volitional symptoms may contribute to the continuous structure of suicide risk in females, as it creates a spectrum of risk rather than distinct categories. However, it should be noted that these interpretations are speculative, as our cross-sectional design captures variation across individuals rather than within individuals over time. Longitudinal research is needed to determine whether the categorical structure in men reflects greater temporal stability and the dimensional structure in women reflects greater fluctuation over time. Second, specific biological and psychosocial factors may contribute to the categorical structure observed in males. For instance, males may have greater impulsivity, higher tolerance for physical pain, or stronger adherence to masculine norms that discourage help-seeking (Richardson et al., 2023; Schrijvers et al., 2012; Sunderland et al., 2023), potentially creating a ‘threshold effect’ where these factors converge to produce a qualitative shift in suicide risk. Future research should investigate these potential mechanisms – including genetic vulnerabilities, dispositional capability for suicide, and cultural factors – to better understand why volitional phase symptoms manifest categorically in males but dimensionally in females.

The present findings suggest that suicide risk assessment and prevention strategies should adopt sex-specific approaches. Furthermore, our results indicate that while distinct high-risk groups for volitional phase symptoms can be identified among males, applying a specific threshold to differentiate groups among females seems artificial. For male outpatients, clinical efforts should focus on the early identification of those approaching or crossing the volitional ‘threshold’ and providing more intensive interventions accordingly. Conversely, for female outpatients, scalable interventions addressing risk factors across all levels are recommended, as the risk appears to accumulate more continuously in this population. Considering that males with more severe volitional phase symptoms have a greater likelihood of death by suicide (Schrijvers et al., 2012), rapidly providing intense interventions to high-risk male patients will prove to be effective for suicide prevention and intervention. However, for female populations, rather than screening for high-risk groups, a more individually tailored treatment approach would be necessary, one that addresses the overall spectrum of suicide risk by sensitively adjusting treatment intensity according to severity, considering individual differences in both motivational and volitional phase symptoms.

## Strengths and limitations

This study had some limitations that must be acknowledged. First, as our analyses were conducted exclusively with patients with mood disorders, additional validation is needed to determine whether these results replicate in the general population and groups with other psychiatric disorders. Second, to meet the data requirements for taxometric analysis, we incorporated reports of suicidal ideation and behavior from various time points as taxometric indicators. Third, we were unable to include objective lethality of suicide attempts as an indicator, which was one of the key taxometric indicators in previous research. Subsequent studies could incorporate this measure as a volitional risk factor to replicate our findings. Fourth, several clinical characteristics that may influence the latent structure of suicide risk – such as the presence of psychotic features, cognitive impairments, illness severity (e.g., age at onset, illness duration), and comorbidities (particularly borderline personality disorder) – were not assessed in the current study. Given that these factors could differentially affect suicide risk in depressive disorders and bipolar disorders, future research should recruit more clinically homogeneous subgroups to re-examine whether the latent structure of suicide risk varies across these characteristics. Furthermore, given that hormonal factors and life stages may influence suicide risk, future research with larger samples should examine whether the latent structure of suicide risk varies across different developmental stages (e.g., adolescence, reproductive years, menopause). Finally, because of the cross-sectional design of this study, we could not determine whether the taxon membership for volitional risk identified in males predicts future suicidal behavior. Longitudinal research is needed to verify whether this taxon membership demonstrates prospective predictive utility (Rufino et al., 2018; Witte et al., 2017).

Despite these limitations, this study has significant clinical implications as the first to examine the latent structure of both motivational and volitional phase symptoms of suicidal behavior within the ideation-to-action framework, while also observing sex differences in the latent structure of suicide risk. These findings suggest the necessity of sex-specific approaches in detecting progression from suicidal ideation to attempts. Regarding intervention, empirical evidence supports the identification of high-risk groups for suicidal behavior enaction among males, whereas for females, a broader perspective addressing various points throughout the suicidal process appears warranted.

## Conclusions

Motivational phase symptoms showed a dimensional structure in females, indicating quantitative rather than qualitative differences in suicidal ideation severity. Males showed ambiguous results for motivational symptoms. For volitional phase symptoms, males exhibited a categorical structure, supporting the approach which classifies individuals into high- or low-risk for enacting suicidal behavior in males, while females showed a dimensional structure. These findings reveal sex differences in both motivational and volitional phase symptoms, highlighting the need for sex-specific approaches when applying the ideation-to-action framework in suicide prevention.

## Supporting information

10.1017/S0033291726104255.sm001 Park et al. supplementary material Park et al. supplementary material

## Supplementary material

The supplementary material for this article can be found at http://doi.org/10.1017/S0033291726104255.
