# Supplementary material for: Sex differences in the latent structure of suicide risk among patients with mood disorders: taxometric analyses using the ideation-to-action framework
Source: Psychol Med. 2026 Apr 28;56:e122. doi: 10.1017/S0033291726104255 (PMC13125938; doi:10.1017/S0033291726104255)
Supplement: Park et al. supplementary material [file S0033291726104255sup001.docx]

**Sex Differences in the Latent Structure of Suicide Risk Among Patients with Mood Disorders: Taxometric Analyses Using the Ideation-to-Action Framework**

Chanhee Park, M.A., Eunbyeol Lee, Ph.D, Myeongkeun Cho, M.A., C. Hyung Keun Park, M.D., Ph.D.

**Contents**

TABLE S1. Taxometric indicators in each sample 3

TABLE S2. Comparison between complement and taxon groups in male group 4

FIGURE S1. Comparison curve fit index (CCFI) profile analysis using motivational indicators conducted on male outpatients 5

FIGURE S2. Comparison curve fit index (CCFI) profile analysis using motivational indicators conducted on female outpatients 6

FIGURE S3. Comparison curve fit index (CCFI) profile analysis using volitional indicators conducted on male outpatients 7

FIGURE S4. Comparison curve fit index (CCFI) profile analysis using volitional indicators conducted on female outpatients 8

FIGURE S5. Three taxometric analyses using motivational indicators conducted on the outpatients with depressive disorders 9

FIGURE S6. Three taxometric analyses using motivational indicators conducted on the outpatients with bipolar disorders 10

FIGURE S7. Three taxometric analyses using volitional indicators conducted on the outpatients with depressive disorders 11

FIGURE S8. Three taxometric analyses using volitional indicators conducted on the outpatients with bipolar disorders 12

FIGURE S9. Comparison curve fit index (CCFI) profile analysis using motivational indicators conducted on outpatients with depressive disorders 13

FIGURE S10. Comparison curve fit index (CCFI) profile analysis using motivational indicators conducted on outpatients with bipolar disorders 14

FIGURE S11. Comparison curve fit index (CCFI) profile analysis using volitional indicators conducted on outpatients with depressive disorders 15

FIGURE S12. Comparison curve fit index (CCFI) profile analysis using volitional indicators conducted on outpatients with bipolar disorders 16

FIGURE S13. Three taxometric analyses using motivational indicators conducted on age- and sample size-matched female outpatient subsamples 17

FIGURE S14. Three taxometric analyses using volitional indicators conducted on age- and sample size-matched female outpatient subsamples 18

FIGURE S15. Comparison curve fit index (CCFI) profile analysis using motivational indicators conducted on age- and sample size-matched female outpatient subsamples 19

FIGURE S16. Comparison curve fit index (CCFI) profile analysis using volitional indicators conducted on age- and sample size-matched female outpatient subsamples 20

Results. Detailed results of sensitivity analyses 21

| Supplementary Table 1. Taxometric indicators in each sample | | | |
| --- | --- | --- | --- |
| Indicator | Cohen’s *d* | Skewness | Kurtosis |
| Male (*N* = 384), motivational indicators | | | |
| Current suicidal ideation | 2.08 | 0.15 | −1.43 |
| Relief | 2.49 | 0.30 | −1.35 |
| Suicidal ideation duration | 1.33 | 0.72 | −0.36 |
| Male (*N* = 340), volitional indicators | | | |
| Current deterrents of suicidal behavior | 1.61 | 1.06 | 0.48 |
| Current suicide planning | 1.49 | 0.49 | −0.62 |
| Lifetime number of suicide attempts | 1.53 | 1.59 | 1.80 |
| Female (*N* = 668), motivational indicators | | | |
| Current suicidal ideation | 2.12 | −0.22 | −1.43 |
| My death would be a relief | 2.47 | 0.14 | −1.50 |
| Suicidal ideation duration | 1.76 | 0.31 | −1.04 |
| Female (*N* = 668), volitional indicators | | | |
| Current deterrents of suicidal behavior | 1.43 | 0.97 | 0.37 |
| Current suicide planning | 1.55 | 0.24 | −0.91 |
| Lifetime number of suicide attempts | 1.64 | 1.10 | 0.08 |

| Supplementary Table 2. Comparison between complement and taxon groups in male group | | | | | | | |
| --- | --- | --- | --- | --- | --- | --- | --- |
|  | Complement Group  (*N* = 122) | | Taxon Group  (*N* = 262) | |  |  |  |
| Variable | *M* | *SD* | *M* | *SD* | *t* | *p* | Effect Size |
| Current depression/anxiety symptoms | | | | | | | |
| Depressive symptoms | 13.11 | 7.12 | 17.82 | 6.48 | −6.43^***^ | <.001 | 0.70 |
| Anxiety symptoms^a^ | 11.31 | 6.28 | 13.33 | 5.32 | −3.07^**^ | .002 | 0.36 |
| Early maladaptive schemas | | | | | | | |
| Social isolation/alienation | 14.46 | 6.35 | 18.63 | 6.53 | −5.87^***^ | <.001 | 0.64 |
| Defectiveness/shame^a^ | 12.26 | 6.22 | 17.28 | 7.22 | −6.99^***^ | <.001 | 0.73 |
| Failure | 12.93 | 6.80 | 16.47 | 7.27 | −4.53^***^ | <.001 | 0.50 |
| Dependence/incompetence | 13.25 | 5.96 | 16.42 | 6.31 | −4.68^***^ | <.001 | 0.51 |
| Suicide risk factors | | | | | | | |
| Perceived burdensomeness^a^ | 12.30 | 7.76 | 22.79 | 10.95 | −10.76^***^ | <.001 | 1.04 |
| Thwarted belongingness | 35.50 | 10.32 | 41.63 | 10.94 | −5.20^***^ | <.001 | 0.57 |
| Fearlessness about death | 13.70 | 6.39 | 15.73 | 7.09 | −2.69^**^ | .004 | 0.30 |
| *Note*. Effect size is Cohen’s *d*. ^**^*p* < .01. ^***^*p* < .001  ^a^*t-*test performed for unequal variances. | | | | | | | |


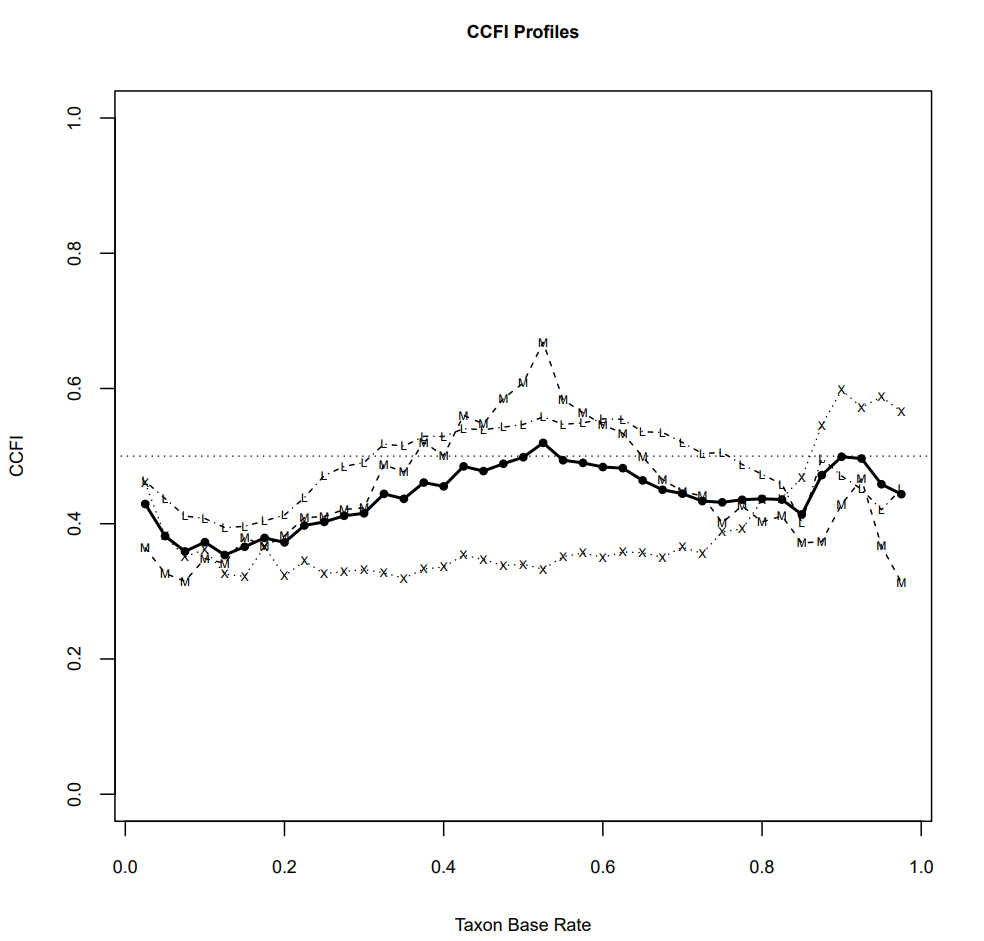


Supplementary Figure 1. Comparison curve fit index (CCFI) profile analysis using motivational indicators conducted on male outpatients. MAMBAC (Mean Above Minus Below A Cut), MAXEIG (MAXimum EIGenvalue), and L-Mode (Latent Mode) taxometric analyses are denoted by the M, X, and L lines, respectively. The composite mean of the CCFI values derived from all three analyses is represented by the darker solid line.


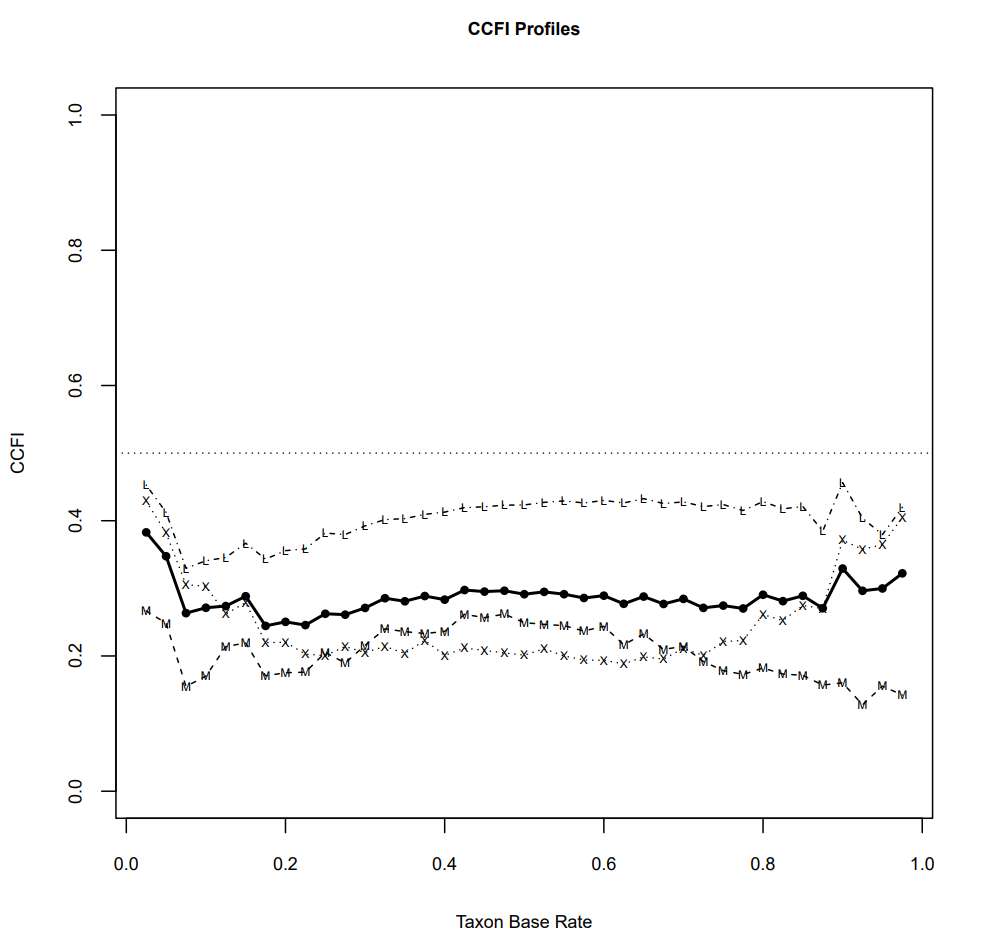


Supplementary Figure 2. Comparison curve fit index (CCFI) profile analysis using motivational indicators conducted on female outpatients. MAMBAC (Mean Above Minus Below A Cut), MAXEIG (MAXimum EIGenvalue), and L-Mode (Latent Mode) taxometric analysis are denoted by M, X, and L lines, respectively. The composite mean of CCFI values derived from all three analyses is represented by the darker, solid s.


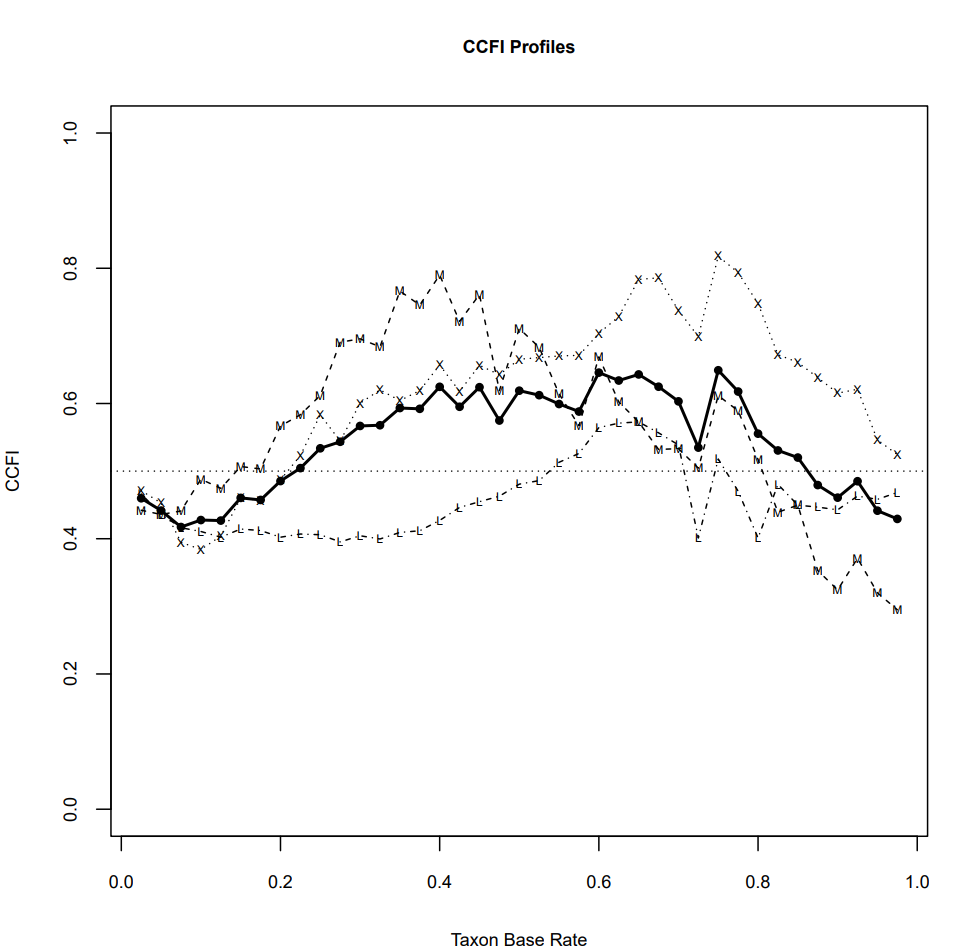


Supplementary Figure 3. Comparison curve fit index (CCFI) profile analysis using volitional indicators conducted on male outpatients. MAMBAC (Mean Above Minus Below A Cut), MAXEIG (MAXimum EIGenvalue), and L-Mode (Latent Mode) taxometric analysis are denoted by M, X, and L lines, respectively. The composite mean of CCFI values derived from all three analyses is represented by the darker, solid line.


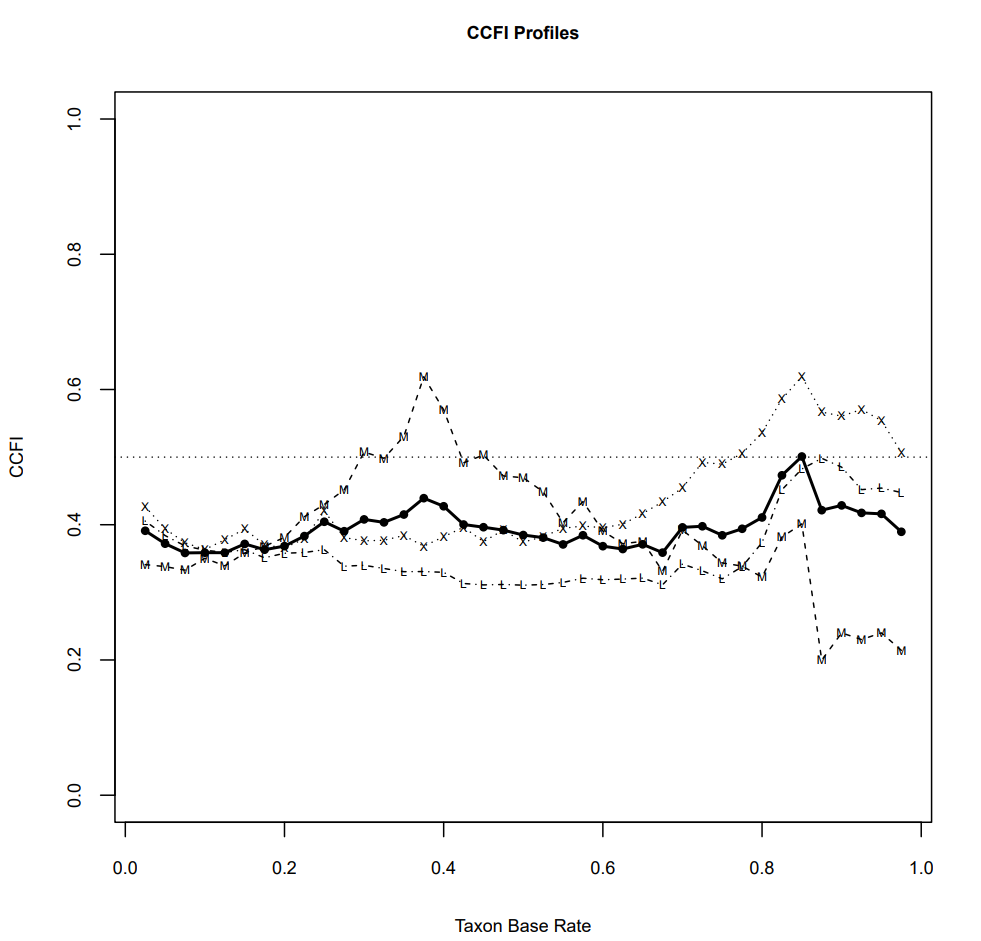


Supplementary Figure 4. Comparison curve fit index (CCFI) profile analysis using volitional indicators conducted on female outpatients. MAMBAC (Mean Above Minus Below A Cut), MAXEIG (MAXimum EIGenvalue), and L-Mode (Latent Mode) taxometric analysis are denoted by M, X, and L lines, respectively. The composite mean of CCFI values derived from all three analyses is represented by the darker, solid line.


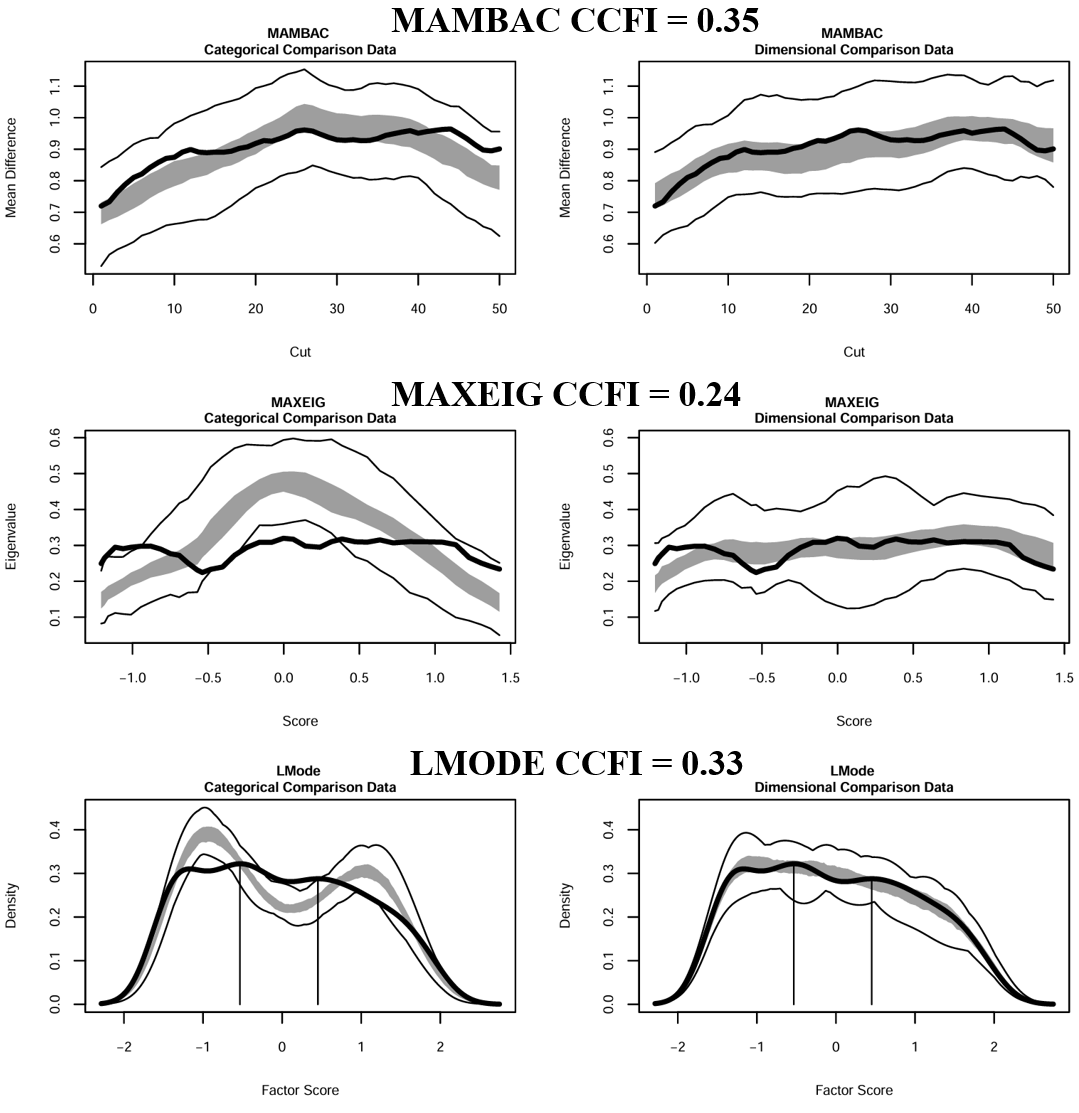


Supplementary Figure 5. Three taxometric analyses using motivational indicators conducted on the outpatients with depressive disorders. Empirical data curves are represented by dark lines, while the boundaries of the comparative analyses are delineated by lighter lines, derived from 100 parallel comparison data samples. The interquartile range (middle 50%) of the values obtained from the parallel comparison data analyses is indicated by the shaded regions. CCFI = comparison curve fit index; MAMBAC = Mean Above Minus Below A Cut; MAXEIG = MAXimum EIGenvalue; L-Mode = latent mode.


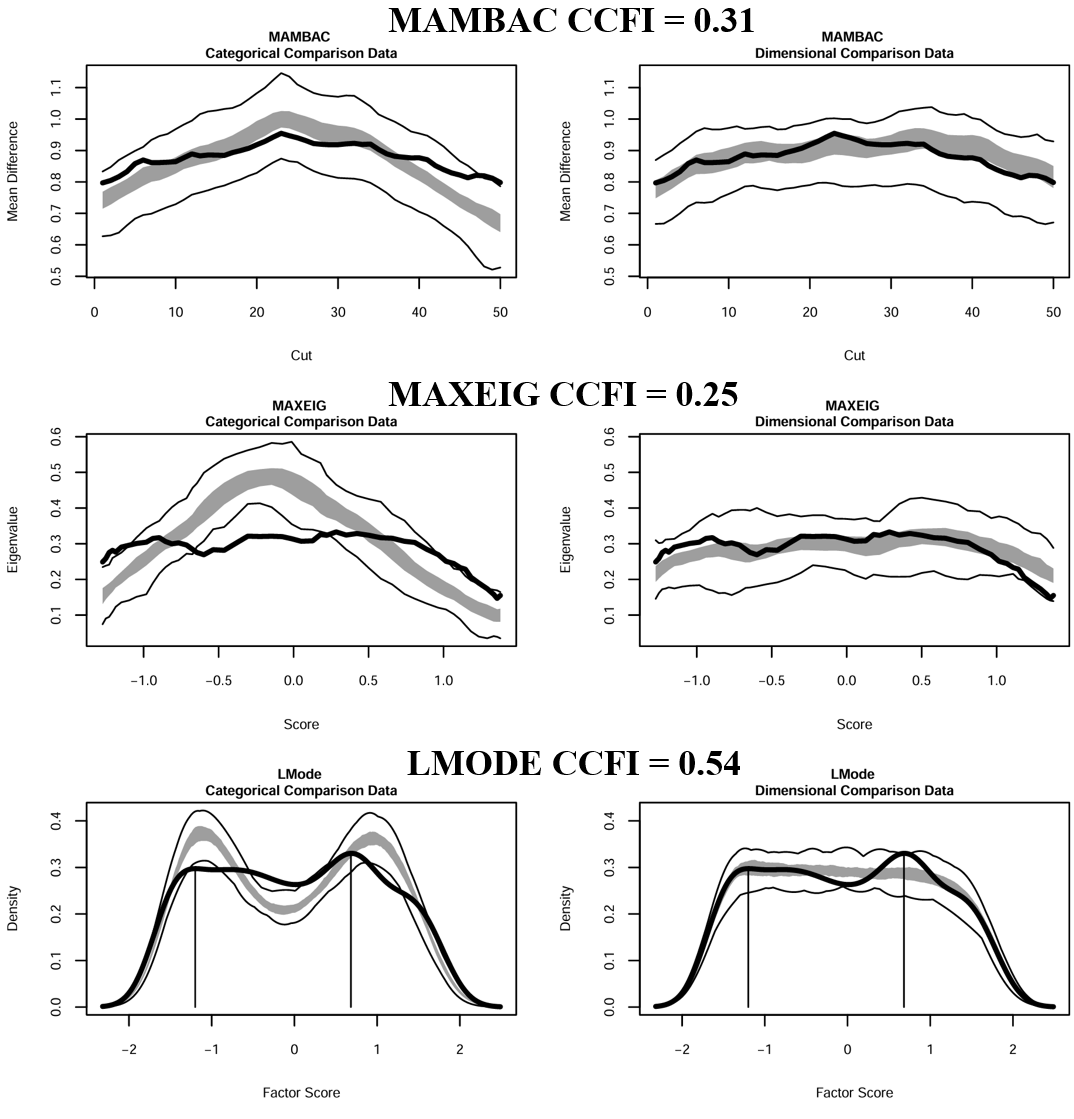


Supplementary Figure 6. Three taxometric analyses using motivational indicators conducted on the outpatients with bipolar disorders. Empirical data curves are represented by dark lines, while the boundaries of the comparative analyses are delineated by lighter lines, derived from 100 parallel comparison data samples. The interquartile range (middle 50%) of the values obtained from the parallel comparison data analyses is indicated by the shaded regions. CCFI = comparison curve fit index; MAMBAC = Mean Above Minus Below A Cut; MAXEIG = MAXimum EIGenvalue; L-Mode = latent mode.


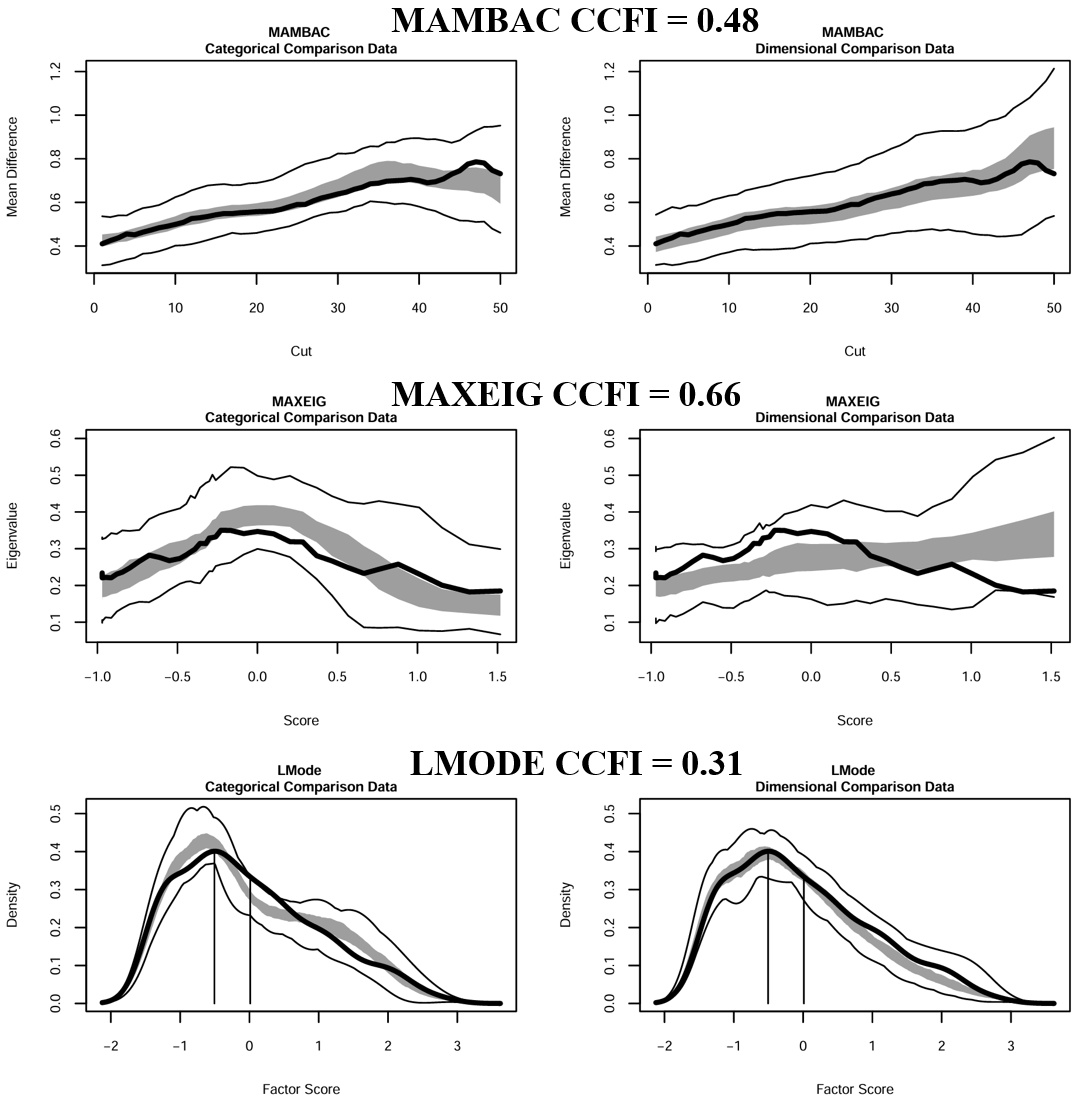


Supplementary Figure 7. Three taxometric analyses using volitional indicators conducted on the outpatients with depressive disorders. Empirical data curves are represented by dark lines, while the boundaries of the comparative analyses are delineated by lighter lines, derived from 100 parallel comparison data samples. The interquartile range (middle 50%) of the values obtained from the parallel comparison data analyses is indicated by the shaded regions. CCFI = comparison curve fit index; MAMBAC = Mean Above Minus Below A Cut; MAXEIG = MAXimum EIGenvalue; L-Mode = latent mode.


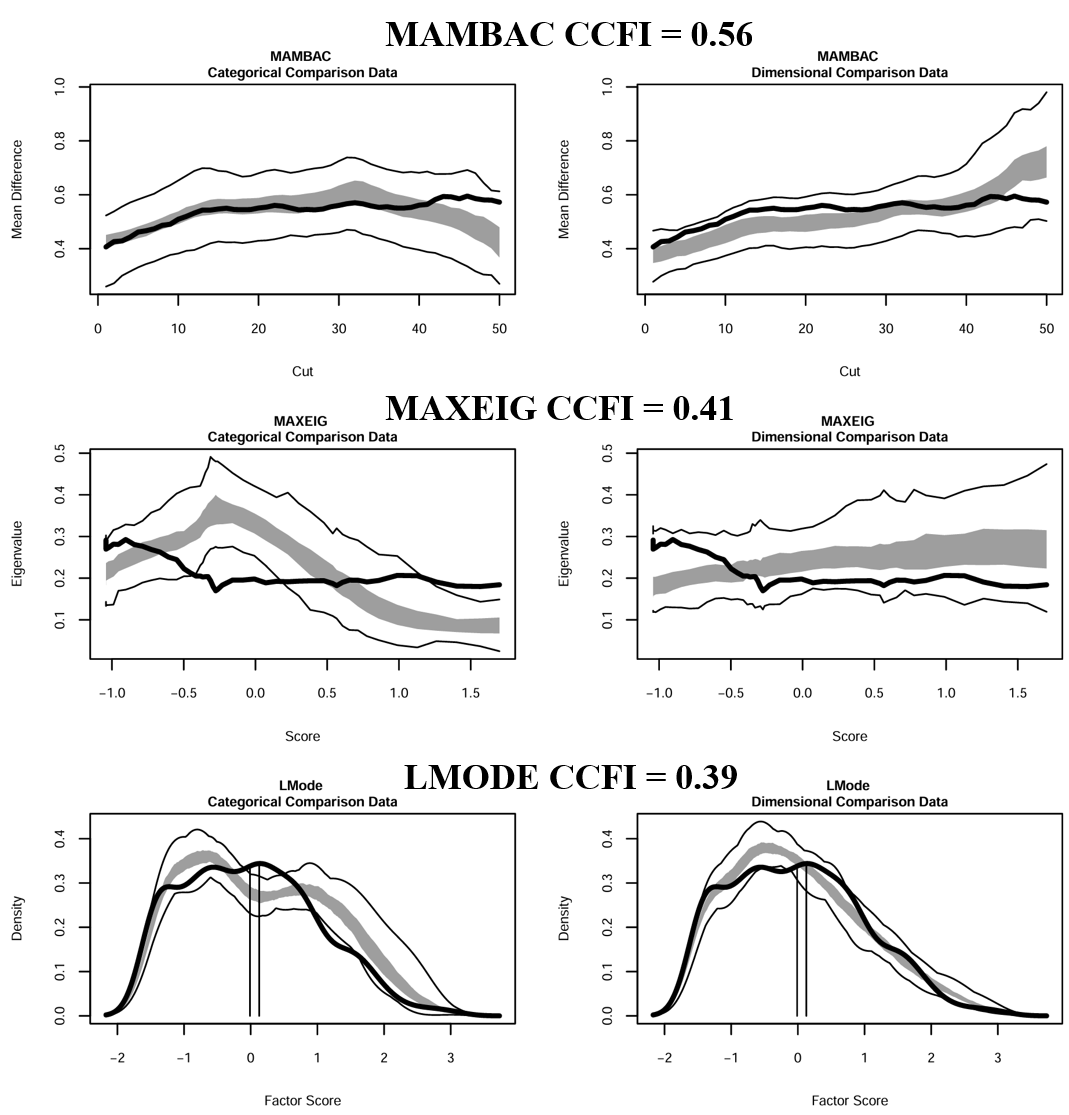


Supplementary Figure 8. Three taxometric analyses using volitional indicators conducted on the outpatients with bipolar disorders. Empirical data curves are represented by dark lines, while the boundaries of the comparative analyses are delineated by lighter lines, derived from 100 parallel comparison data samples. The interquartile range (middle 50%) of the values obtained from the parallel comparison data analyses is indicated by the shaded regions. CCFI = comparison curve fit index; MAMBAC = Mean Above Minus Below A Cut; MAXEIG = MAXimum EIGenvalue; L-Mode = latent mode.


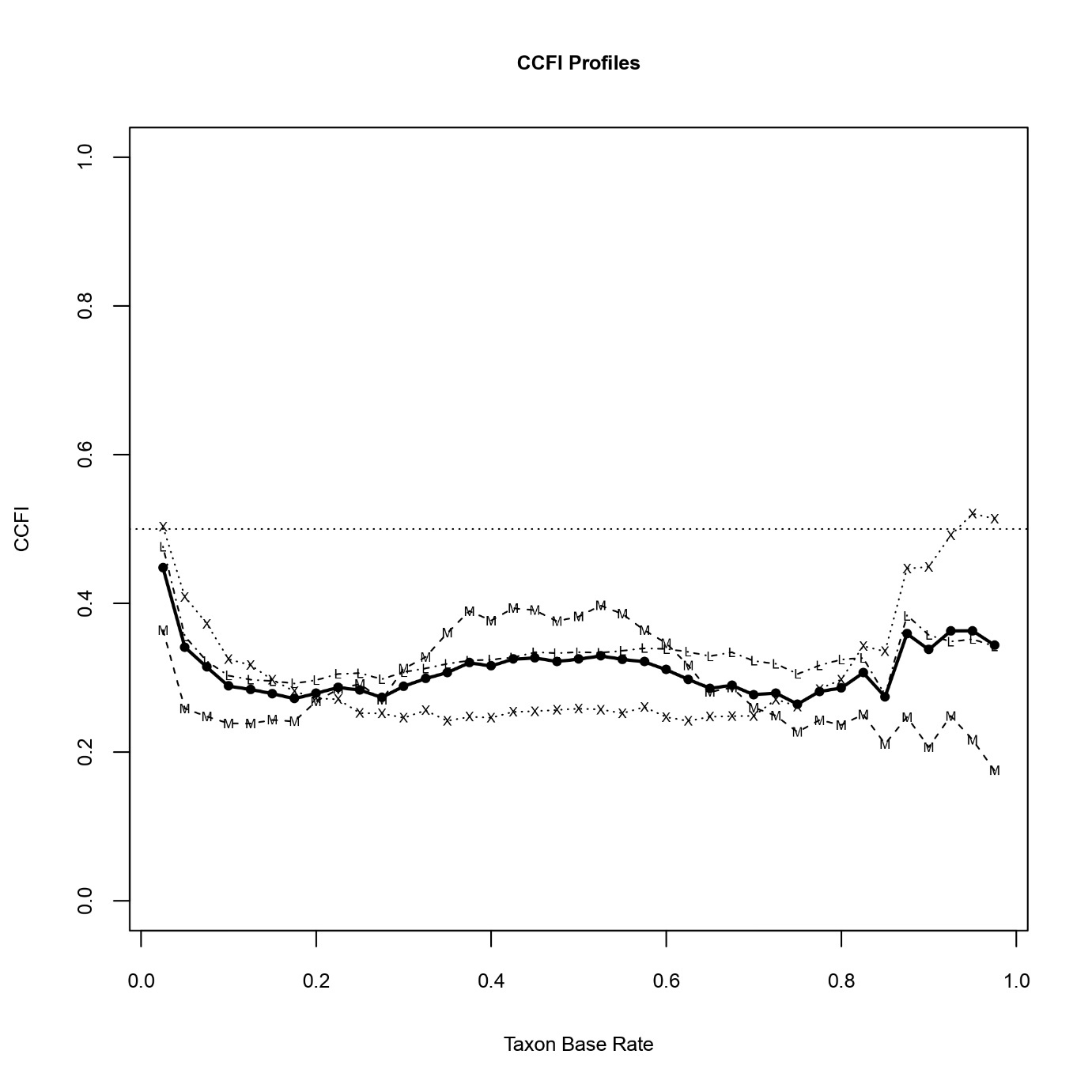


Supplementary Figure 9. Comparison curve fit index (CCFI) profile analysis using motivational indicators conducted on outpatients with depressive disorders. MAMBAC (Mean Above Minus Below A Cut), MAXEIG (MAXimum EIGenvalue), and L-Mode (Latent Mode) taxometric analysis are denoted by M, X, and L lines, respectively. The composite mean of CCFI values derived from all three analyses is represented by the darker, solid line.


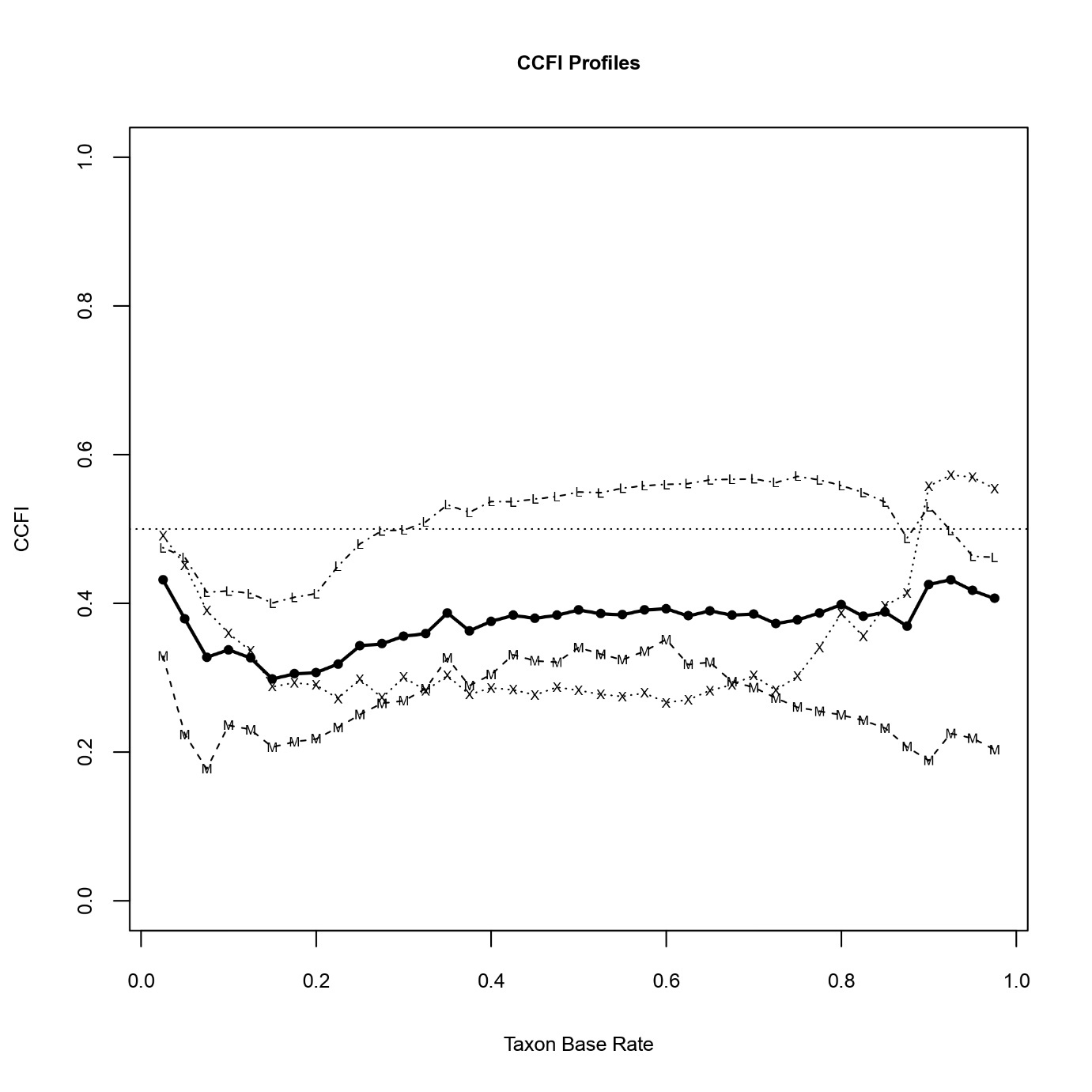


Supplementary Figure 10. Comparison curve fit index (CCFI) profile analysis using motivational indicators conducted on outpatients with bipolar disorders. MAMBAC (Mean Above Minus Below A Cut), MAXEIG (MAXimum EIGenvalue), and L-Mode (Latent Mode) taxometric analysis are denoted by M, X, and L lines, respectively. The composite mean of CCFI values derived from all three analyses is represented by the darker, solid line.


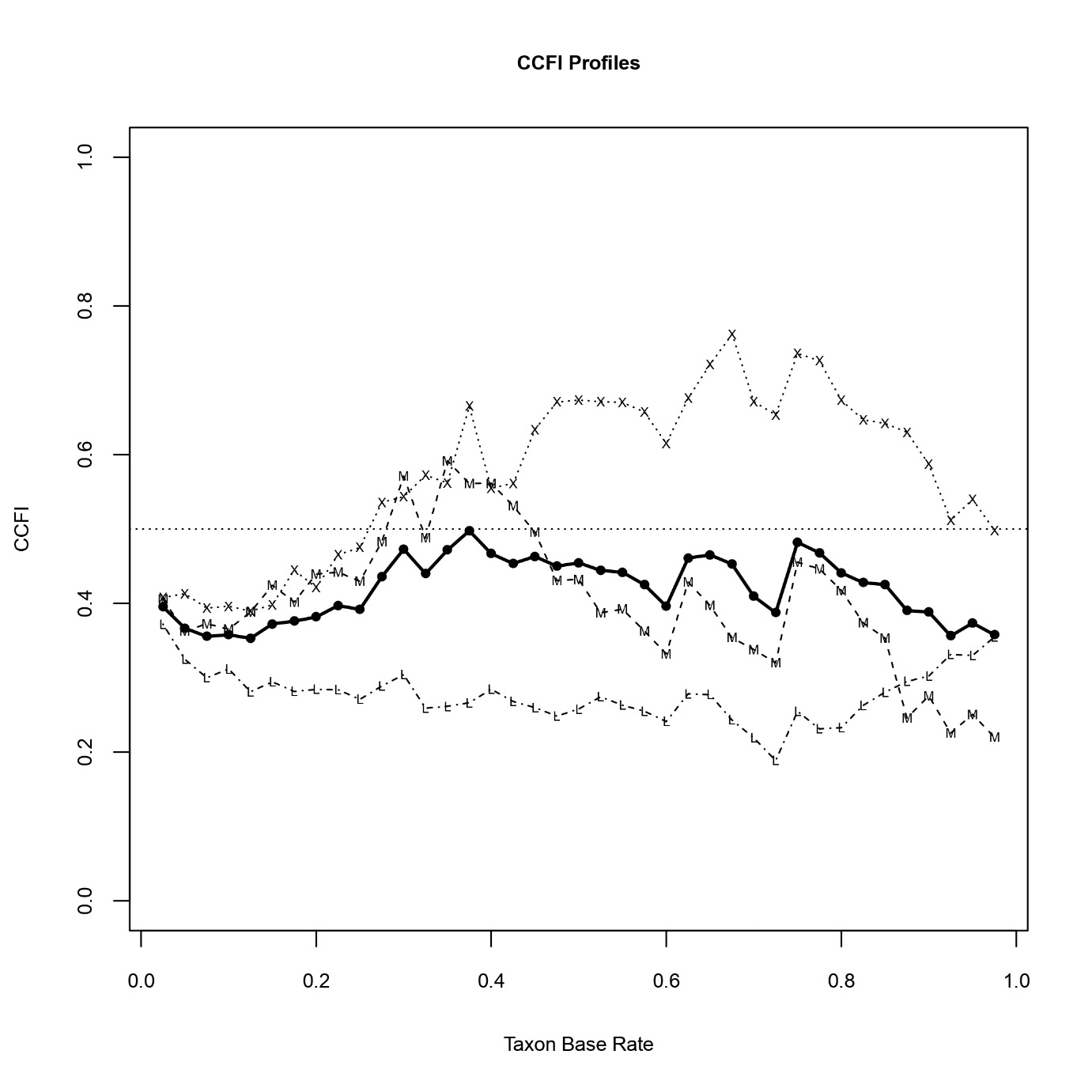


Supplementary Figure 11. Comparison curve fit index (CCFI) profile analysis using volitional indicators conducted on outpatients with depressive disorders. MAMBAC (Mean Above Minus Below A Cut), MAXEIG (MAXimum EIGenvalue), and L-Mode (Latent Mode) taxometric analysis are denoted by M, X, and L lines, respectively. The composite mean of CCFI values derived from all three analyses is represented by the darker, solid line.


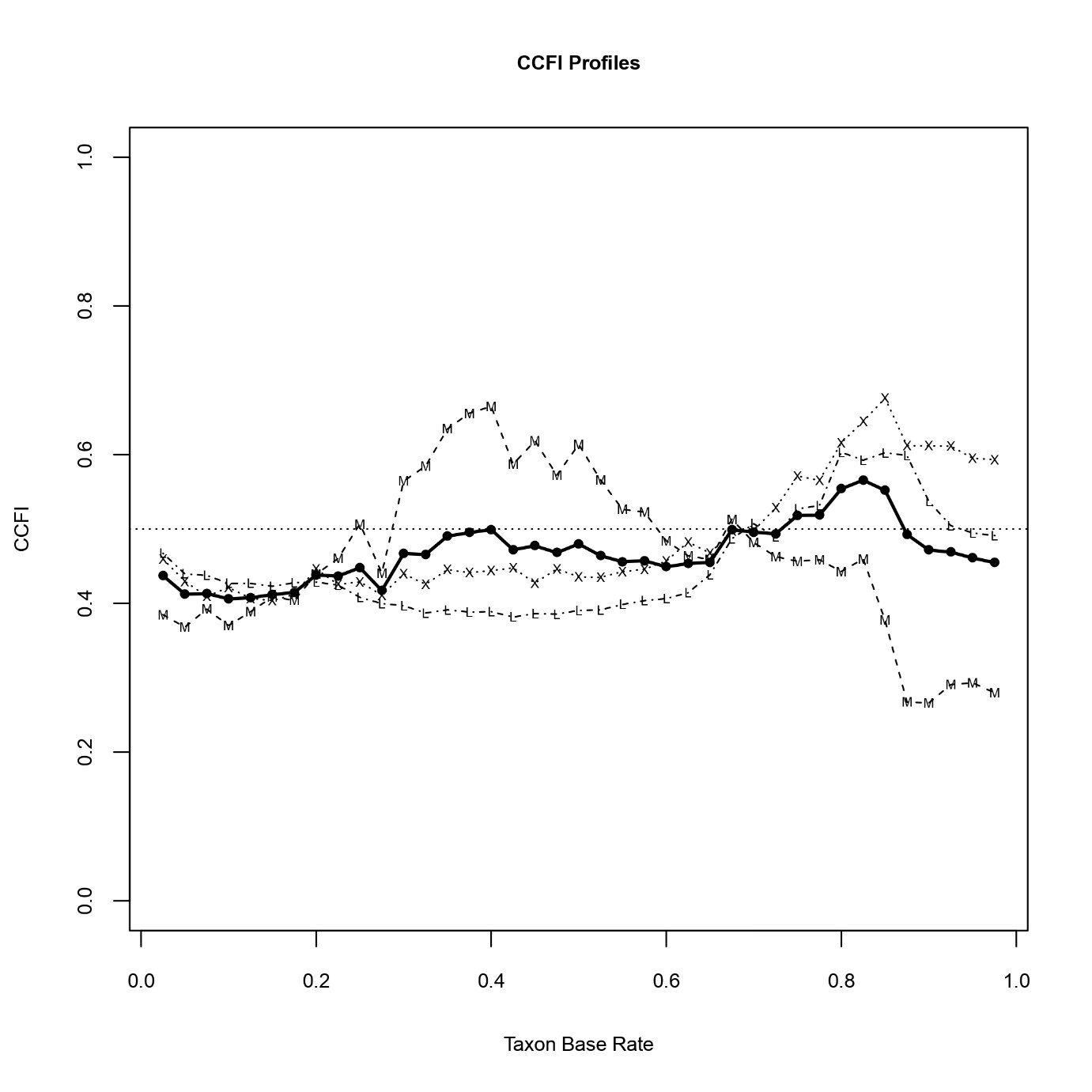


Supplementary Figure 12. Comparison curve fit index (CCFI) profile analysis using volitional indicators conducted on outpatients with bipolar disorders. MAMBAC (Mean Above Minus Below A Cut), MAXEIG (MAXimum EIGenvalue), and L-Mode (Latent Mode) taxometric analysis are denoted by M, X, and L lines, respectively. The composite mean of CCFI values derived from all three analyses is represented by the darker, solid line.


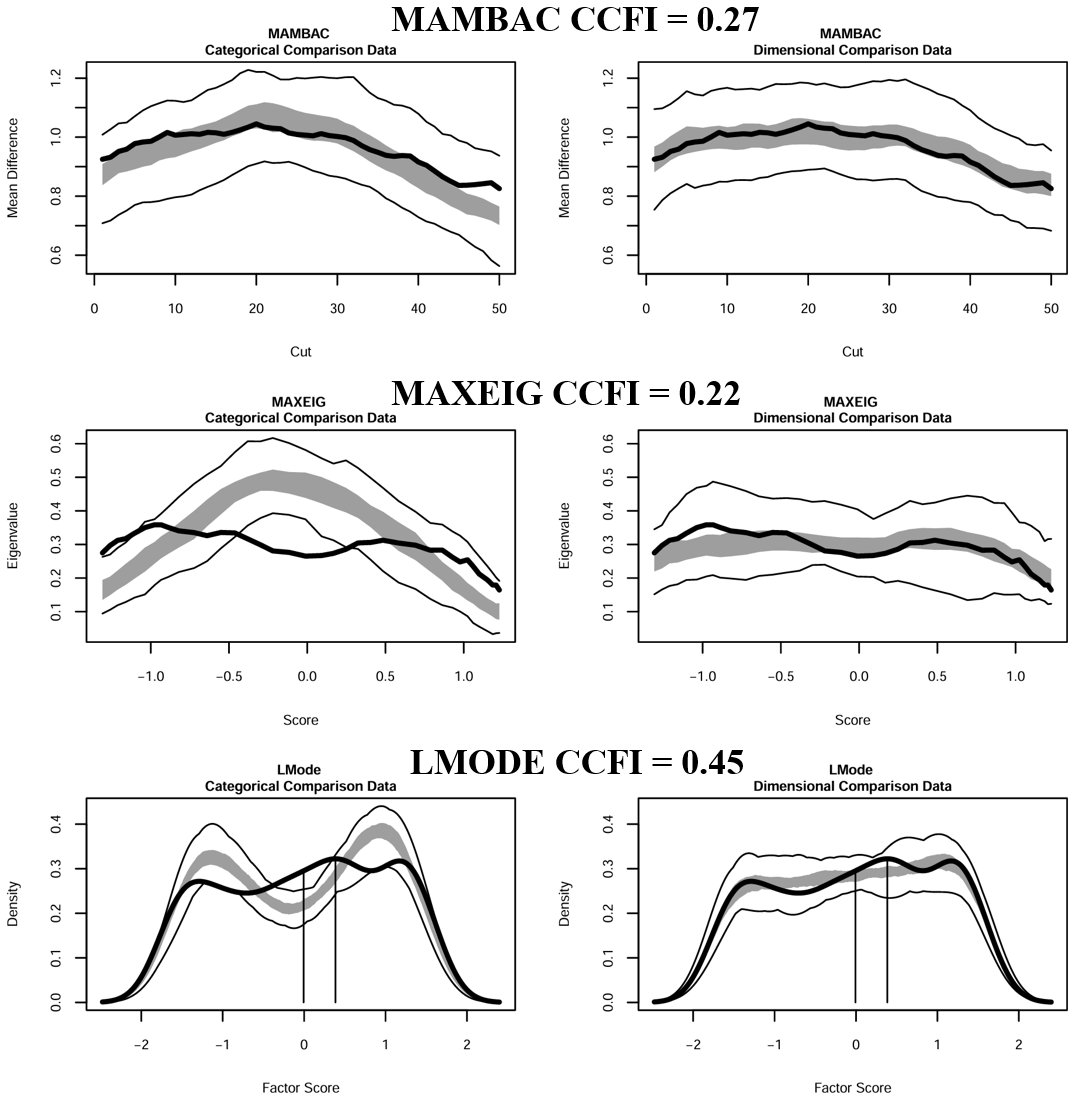


Supplementary Figure 13. Three taxometric analyses using motivational indicators conducted on age- and sample size-matched female outpatient subsamples. Empirical data curves are represented by dark lines, while the boundaries of the comparative analyses are delineated by lighter lines, derived from 100 parallel comparison data samples. The interquartile range (middle 50%) of the values obtained from the parallel comparison data analyses is indicated by the shaded regions. CCFI = comparison curve fit index; MAMBAC = Mean Above Minus Below A Cut; MAXEIG = MAXimum EIGenvalue; L-Mode = latent mode.


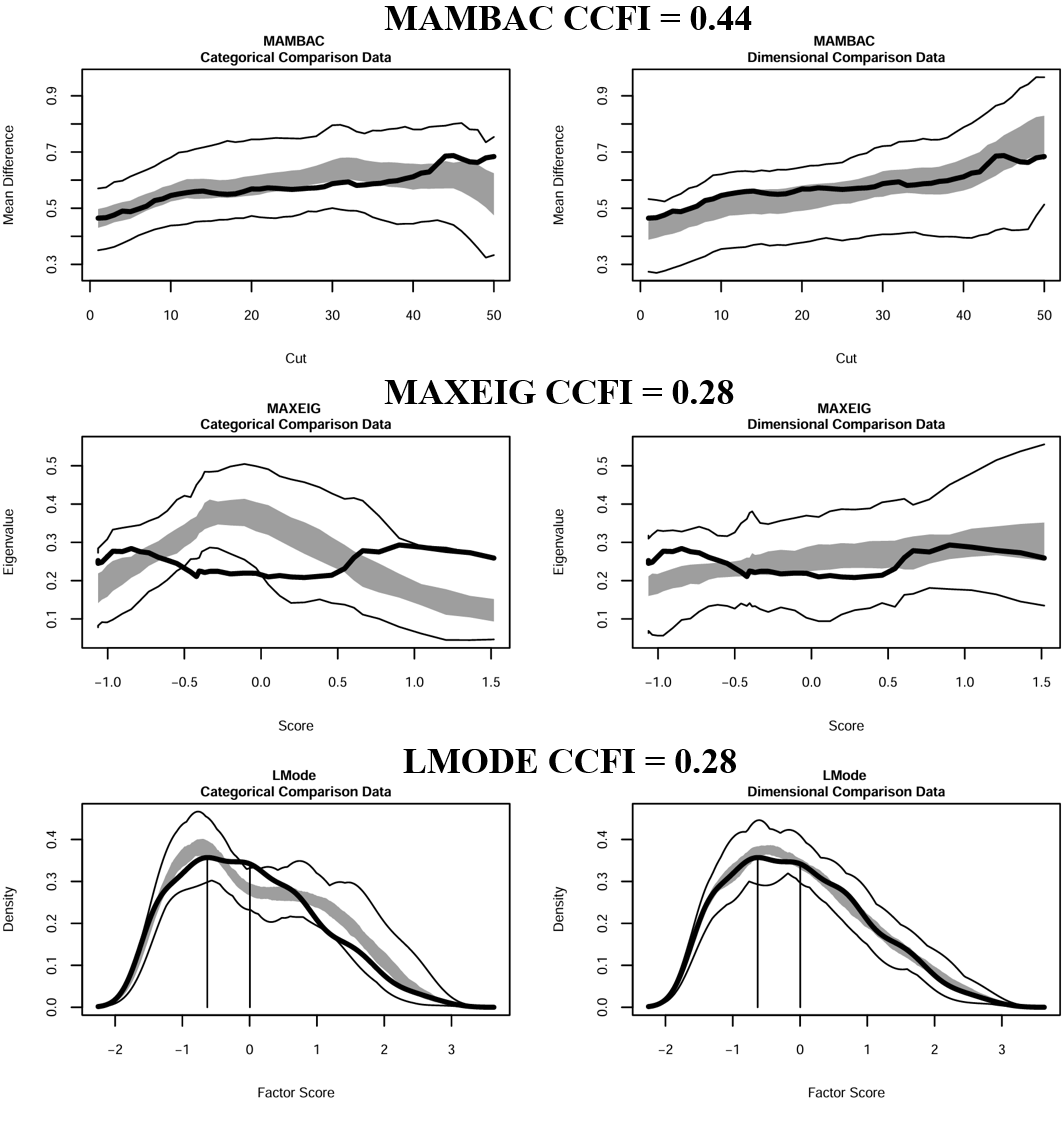


Supplementary Figure 14. Three taxometric analyses using volitional indicators conducted on age- and sample size-matched female outpatient subsamples. Empirical data curves are represented by dark lines, while the boundaries of the comparative analyses are delineated by lighter lines, derived from 100 parallel comparison data samples. The interquartile range (middle 50%) of the values obtained from the parallel comparison data analyses is indicated by the shaded regions. CCFI = comparison curve fit index; MAMBAC = Mean Above Minus Below A Cut; MAXEIG = MAXimum EIGenvalue; L-Mode = latent mode.


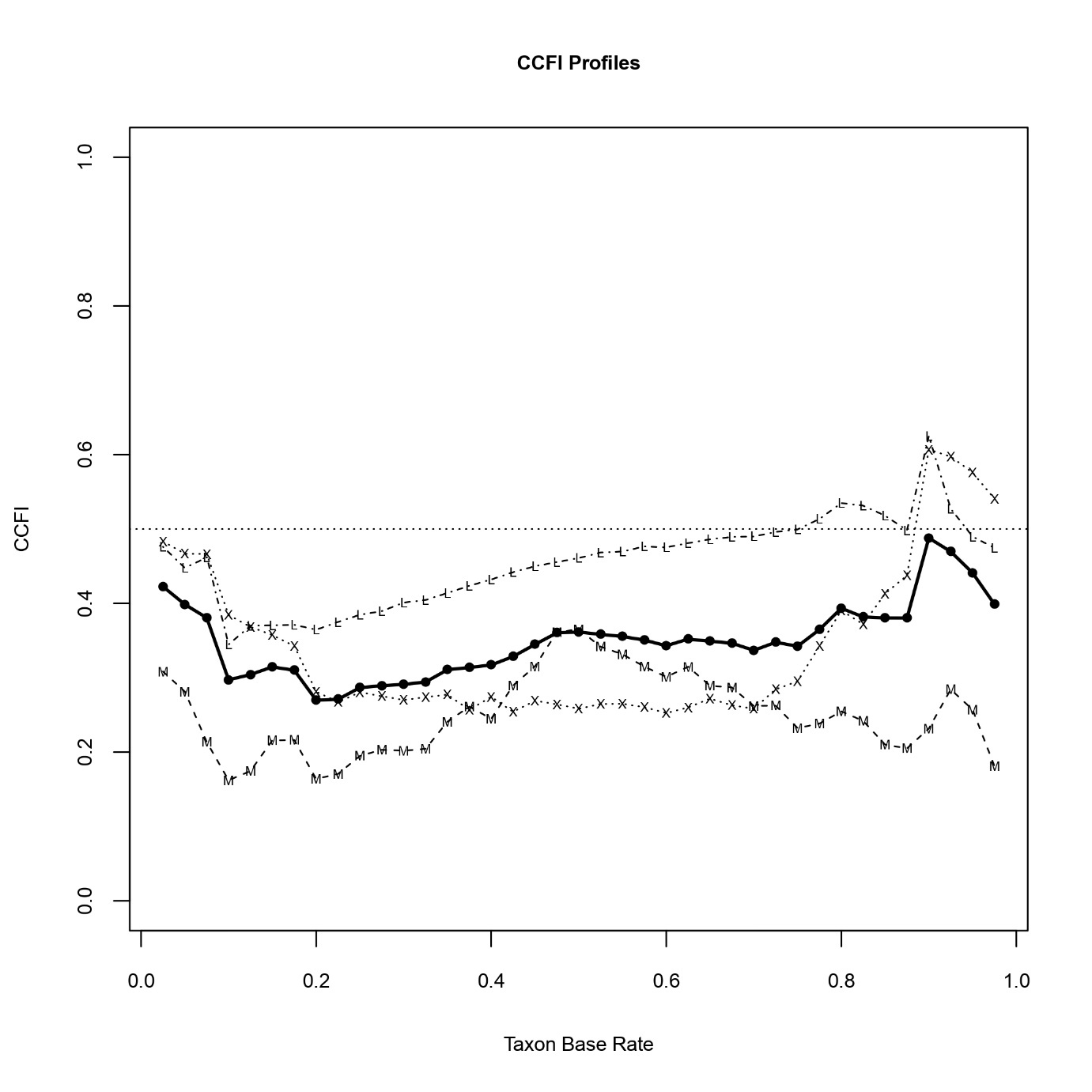


Supplementary Figure 15. Comparison curve fit index (CCFI) profile analysis using motivational indicators conducted on age- and sample size-matched female outpatient subsamples. MAMBAC (Mean Above Minus Below A Cut), MAXEIG (MAXimum EIGenvalue), and L-Mode (Latent Mode) taxometric analysis are denoted by M, X, and L lines, respectively. The composite mean of CCFI values derived from all three analyses is represented by the darker, solid line.


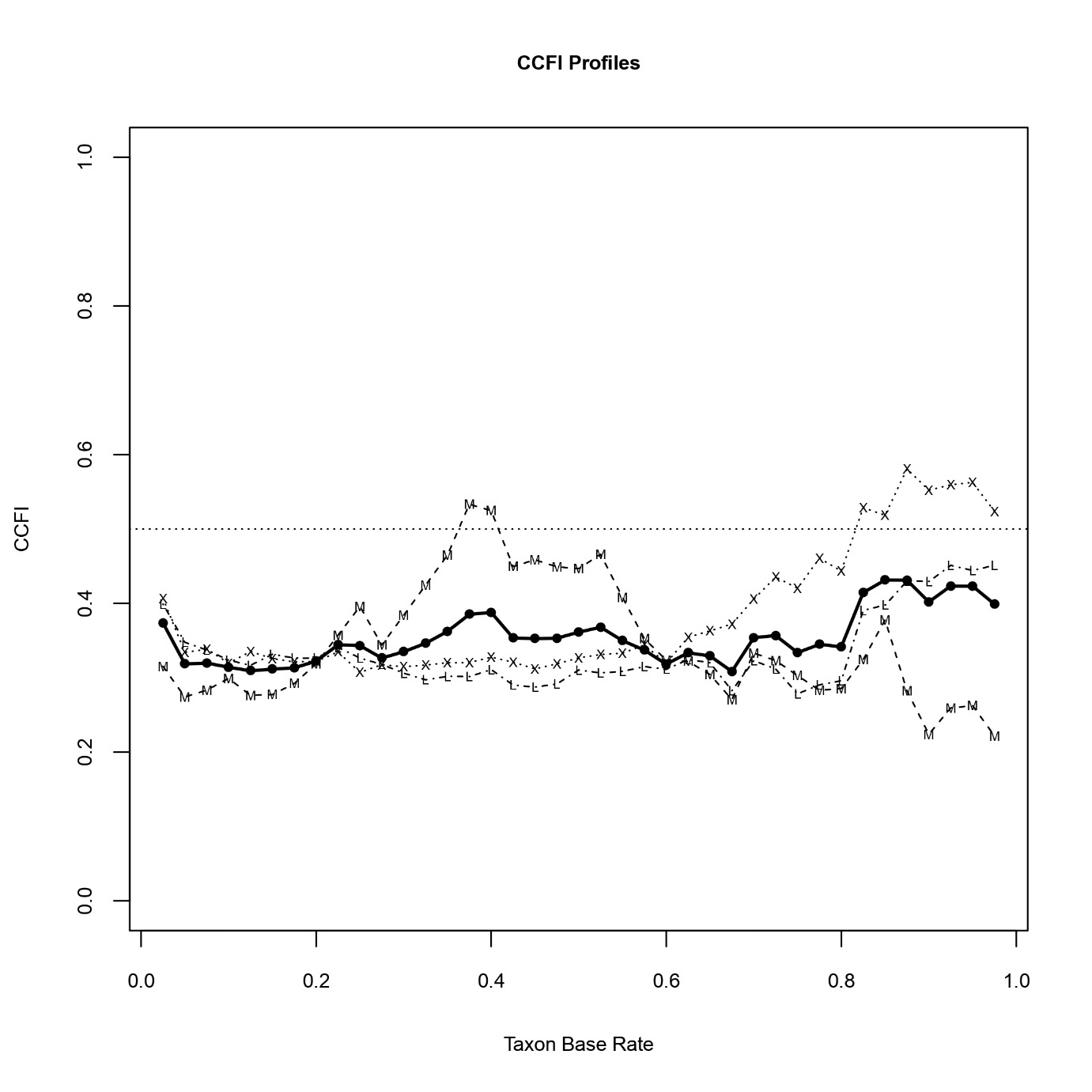


Supplementary Figure 16. Comparison curve fit index (CCFI) profile analysis using volitional indicators conducted on age- and sample size-matched female outpatient subsamples. MAMBAC (Mean Above Minus Below A Cut), MAXEIG (MAXimum EIGenvalue), and L-Mode (Latent Mode) taxometric analysis are denoted by M, X, and L lines, respectively. The composite mean of CCFI values derived from all three analyses is represented by the darker, solid line.

**Detailed results of sensitivity analyses**

**Diagnostic Group Analyses**

Motivational Phase Indicators. For patients with depressive disorders, the results supported dimensionality, with CCFI values of 0.35 (MAMBAC), 0.24 (MAXEIG), and 0.33 (L-Mode), averaging 0.31 (Figure S5). The CCFI profile method yielded 0.31, further supporting the dimensional structure (Figure S9). For patients with bipolar disorders, the findings were consistent, showing CCFI values of 0.31 (MAMBAC), 0.25 (MAXEIG), and 0.54 (L-Mode), averaging 0.36 (Figure S6). The CCFI profile method yielded 0.36, also indicating a dimensional latent structure (Figure S10).

Volitional Phase Indicators. For patients with depressive disorders, the results were ambiguous, with CCFI values of 0.48 (MAMBAC), 0.66 (MAXEIG), and 0.31 (L-Mode), averaging 0.49 (Figure S7). The CCFI profile method yielded 0.44, suggesting an ambiguous latent structure (Figure S11). Similarly, for patients with bipolar disorders, the results remained ambiguous, with CCFI values of 0.56 (MAMBAC), 0.41 (MAXEIG), and 0.39 (L-Mode), averaging 0.45 (Figure S8). The CCFI profile method yielded 0.49, indicating an ambiguous structure (Figure S12).

**Age- and Sample Size-Matched Female Subsample Analyses**

Motivational Phase Indicators. For the matched female subsamples, the results supported dimensionality, with CCFI values of 0.27 (MAMBAC), 0.22 (MAXEIG), and 0.45 (L-Mode), averaging 0.31 (Figure S13). The CCFI profile method yielded 0.36, supporting the dimensional structure (Figure S15).

Volitional Phase Indicators. For the matched female subsamples, the results supported dimensionality, with CCFI values of 0.44 (MAMBAC), 0.28 (MAXEIG), and 0.28 (L-Mode), averaging 0.34 (Figure S14). The CCFI profile method yielded 0.36, indicating a dimensional latent structure (Figure S16).
